# Supplementary material for: A dodecamethoxy[6]cycloparaphenylene consisting entirely of hydroquinone ethers: unveiling in-plane aromaticity through a rotaxane structure
Source: Nat Commun. 2023 Dec 7;14:8091. doi: 10.1038/s41467-023-43907-7 (PMC10703805; doi:10.1038/s41467-023-43907-7)
Supplement: Supplementary file 1 — Supplementary Information [file 41467_2023_43907_MOESM1_ESM.pdf]

Supplementary Information of

A Dodecamethoxy[6]cycloparaphenylene Consisting Entirely of Hydroquinone Ethers: Unveiling In-plane Aromaticity Through a Rotaxane Structure

Naoki Narita,[a]† Yusuke Kurita,[a]† Kohtaro Osakada,[b] Tomohito Ide,*[c] Hidetoshi Kawai,*[a] and Yoshitaka Tsuchido*[a]

[a] Department of Chemistry, Faculty of Science, Tokyo University of Science, 1–3 Kagurazaka, Shinjuku-ku, Tokyo 162-8601, Japan

E-mail: tsuchido@rs.tus.ac.jp, kawaih@rs.tus.ac.jp

[b] Laboratory for Chemistry and Life Science, Institute of Innovative Research, Tokyo Institute of Technology, 4259, Nagatsuta, Midori-ku, Yokohama 226-8503, Japan

[c] Department of Chemical Science and Engineering, National Institute of Technology, Tokyo College, 1220-2 Kunugida-machi, Hachioji-shi, Tokyo, 193-0997, Japan

E-mail: ide@tokyo-ct.ac.jp

† N.N. and Y.K. contributed equally.

Table of contents

Supplementary Figures and Tables 4

Supplementary Fig. 1 Synthetic routes for [6]CPP-12OMe (**1**). 4

Supplementary Fig. 2 ^{1}H NMR spectrum of macrocyclic Au complex (**Au-1**). 5

Supplementary Fig. 3 ^{31}P{^{1}H} NMR spectrum of macrocyclic Au complex (**Au-1**). 5

Supplementary Fig. 4 ^{13}C{^{1}H} NMR spectrum of macrocyclic Au complex (**Au-1**). 6

Supplementary Fig. 5 ^{1}H NMR spectrum of [6]CPP-12OMe (**1**). 6

Supplementary Fig. 6 ^{13}C{^{1}H} NMR spectrum of [6]CPP-12OMe (**1**). 7

Supplementary Fig. 7 HRMS spectrum of [6]CPP-12OMe (**1**). 7

Supplementary Fig. 8 ^{1}H NMR spectrum of [2]rotaxane (**2**). 8

Supplementary Fig. 9 HRMS spectrum of [2]rotaxane (**2**). 8

Supplementary Fig. 10 Optimized structure of [2]rotaxane (**2**) by DFT calculation. 9

Supplementary Fig. 11 ^{1}H NMR spectrum of [2]rotaxane (**2**) upon addition of 2 equiv. of Magic Blue. 10

Supplementary Fig. 12 ^{1}H-^{1}H COSY spectrum of [2]rotaxane (**2**) upon addition of 2 equiv. of Magic Blue. 10

Supplementary Fig. 13 Optimized structures of [2]rotaxane-dication ($\mathbf{2}^{2+}$) with different alkyl chain configuration. 11

Supplementary Fig. 14 Chiral HPLC charts of [6]CPP-12OMe (**1**) and [2]rotaxane (**2**). 12

Supplementary Fig. 15 Variable-temperature ^{1}H NMR spectra of [6]CPP-12OMe (**1**). 12

Supplementary Fig. 16 Phenylene flipping behavior of [6]CPP-12OMe (**1**). 13

Supplementary Fig. 17 Energy diagrams and Kohn-Sham orbitals of [6]CPP and [6]CPP-12OMe (**1**). 14

Supplementary Table 1 Orbital energies of [6]CPP. 15

Supplementary Table 2 Orbital energies of [6]CPP-12OMe (**1**). 15

Supplementary Table 3 Strain energies of [6]CPP and [6]CPP-12OMe (**1**). 15

Supplementary Fig. 18 Bond critical points (BCPs) and bond path analysis of [6]CPP and [6]CPP-12OMe (**1**). 18

Supplementary Fig. 19 Non-Covalent Interaction (NCI) plots of [6]CPP and [6]CPP-12OMe (**1**). 18

Supplementary Fig. 20 ^{1}H NMR titration of [6]CPP-12OMe (**1**) with Magic Blue. 17

Supplementary Table 4 The first five states of calculated transition properties and frontier orbitals (SCF level) of neutral [6]CPP-12OMe. 18

Supplementary Table 5 The first five states of calculated transition properties and frontier orbitals (SCF level) of [6]CPP-12OMe cation radical. 19

Supplementary Table 6 The first five states of calculated transition properties and frontier orbitals (SCF level) of [6]CPP-12OMe dication. 20

Supplementary Fig. 21 Density differences of the first excitation state of neutral, cation radical, and dication species of [6]CPP-12OMe. 21

Supplementary Table 7 List of averaged NICS values of [6]CPP-12OMe (**1**), cation radical ($\mathbf{1}^{+\bullet}$), dication ($\mathbf{1}^{2+}$), and dication of [6]CPP. 21

Supplementary Fig. 22 2D-NICS and ACID plots of dication ($\mathbf{1}^{2+}$). 21
Supplementary Fig. 23 Results of guest inclusion test for [6]CPP-12OMe (**1**). 22
Supplementary Fig. 24 Stacked ^{1}H NMR spectra of [6]CPP-12OMe (**1**) with excess amount of cyanoalkanes. 22
Supplementary Fig. 25 Stacked ^{1}H NMR spectra of [6]CPP-12OMe (**1**) with excess amount of various guest molecules. 23
Supplementary Fig. 26 Stacked ^{1}H NMR spectra of [6]CPP-12OMe (**1**) with excess amount of bulky or electron-deficient guest molecules. 23
Supplementary Fig. 27 ^{1}H NMR titration and titration profile of [6]CPP-12OMe (**1**) with NC-$(CH_2)_4$-CN (adiponitrile, **G1**). 24
Supplementary Fig. 28 UV/vis absorption titration of [6]CPP-12OMe (**1**) with NC-$(CH_2)_4$-CN (adiponitrile, **G1**). 25
Supplementary Fig. 29 Results of guest inclusion test for [6]CPP-12OMe dication ($\mathbf{1}^{2+}$). 26
Supplementary Fig. 30 Stacked ^{1}H NMR spectra of [6]CPP-12OMe dication ($\mathbf{1}^{2+}$) with excess amount of various guest molecules. 26
Supplementary Fig. 31 ORTEP drawings of macrocyclic Au complex (**Au-1** and **Au-1'**). 27
Supplementary Table 8 List of selected atom distances and angles for **Au-1** and **Au-1'**. 27
Supplementary Fig. 32 ORTEP drawing of [6]CPP-12OMe (**1**). 28
Supplementary Fig. 33 ORTEP drawing of [6]CPP-12OMe dication ($\mathbf{1}^{2+}$). 28
Supplementary Table 9 List of averaged bond length and diameters of [6]CPP-12OMe dication ($\mathbf{1}^{2+}$), [6]CPP-12OMe (**1**), [6]CPP, and 1,4-dimthoxybenzene. 29
Supplementary Table 10 List of averaged harmonic oscillator model of aromaticity (HOMA) value of [6]CPP, [6]CPP-12OMe (**1**), and [6]CPP-12OMe dication ($\mathbf{1}^{2+}$). 29
Supplementary Fig. 34 ORTEP drawing of inclusion complex (**1⊃G1**). 30
Supplementary Table 11 Crystal data and structure refinement for macrocyclic Au complex (**Au-1**), [6]CPP-12OMe (**1**), [6]CPP-12OMe dication ($\mathbf{1}^{2+}$), and inclusion complex (**1⊃G1**). 31

Supplementary Methods 32
Supplementary Method 1 Synthesis of $[Au_2Cl_2(dcpm)]$. 32
Supplementary Method 2 Synthesis of 2,2',5,5'-tetramethoxy-1,1'-biphenyl (**4**). 32
Supplementary Method 3 Synthesis of 4,4'-dibromo-2,2',5,5'-tetramethoxybiphenyl (**3**). 32
Supplementary Method 4 Synthesis of (2,2',5,5'-tetramethoxy-[1,1'-biphenyl]-4,4'-diyl)diboronic acid (**L1**). 33
Supplementary Method 5 Crystallographic study. 33
Supplementary Method 6 Computational details. 34

Supplementary Notes 36

Supplementary References 37

Supplementary Figures and Tables

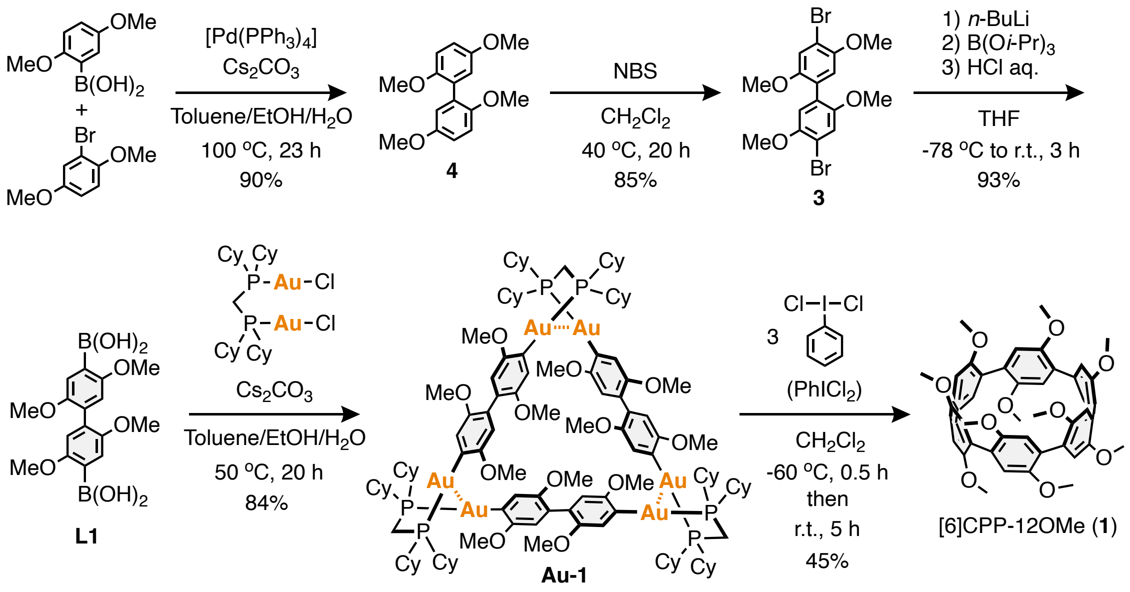

Supplementary Fig. 1 Synthetic routes for [6]CPP-12OMe (**1**) *via* the macrocyclic Au complex. The procedures for the synthesis of the known compounds **4**, **3**, and **L1** were described in the Supplementary Methods 2-4, and those for the new compounds **Au-1** and **1** were described in the Methods section of the manuscript.

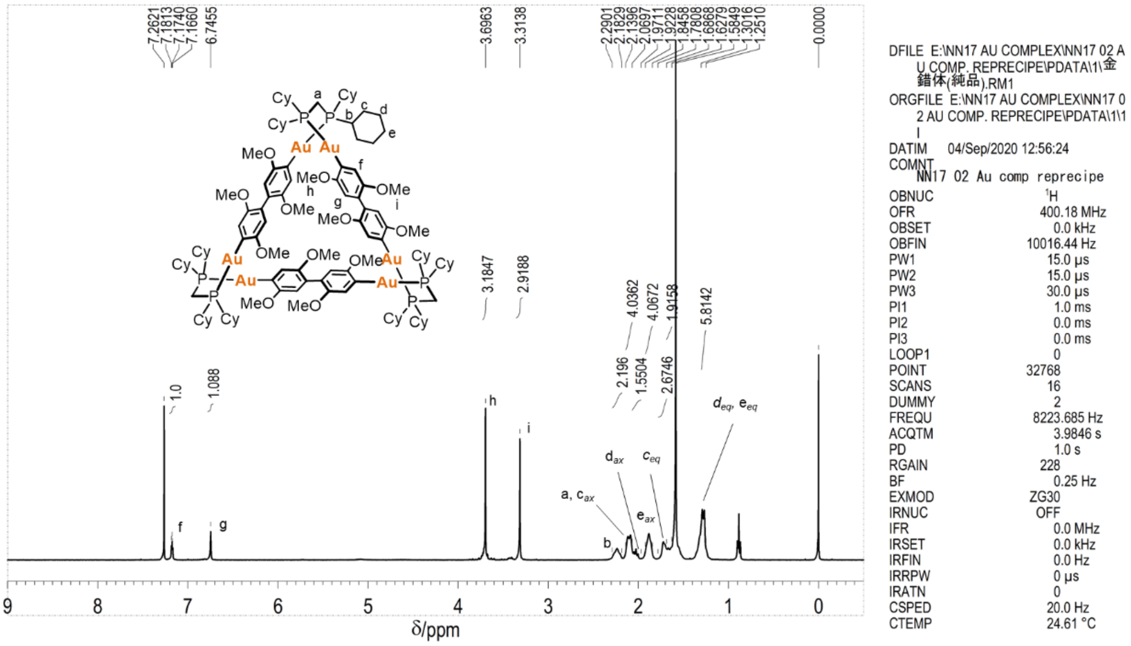

Supplementary Fig. 2 ^{1}H NMR spectrum of macrocyclic Au complex, $[Au_2(C_6H_4\text{-}2,5\text{-}OMe)_2(Cy_2PCH_2PCy_2)]_3$ (**Au-1**) (400 MHz, $CDCl_3$, 297 K).

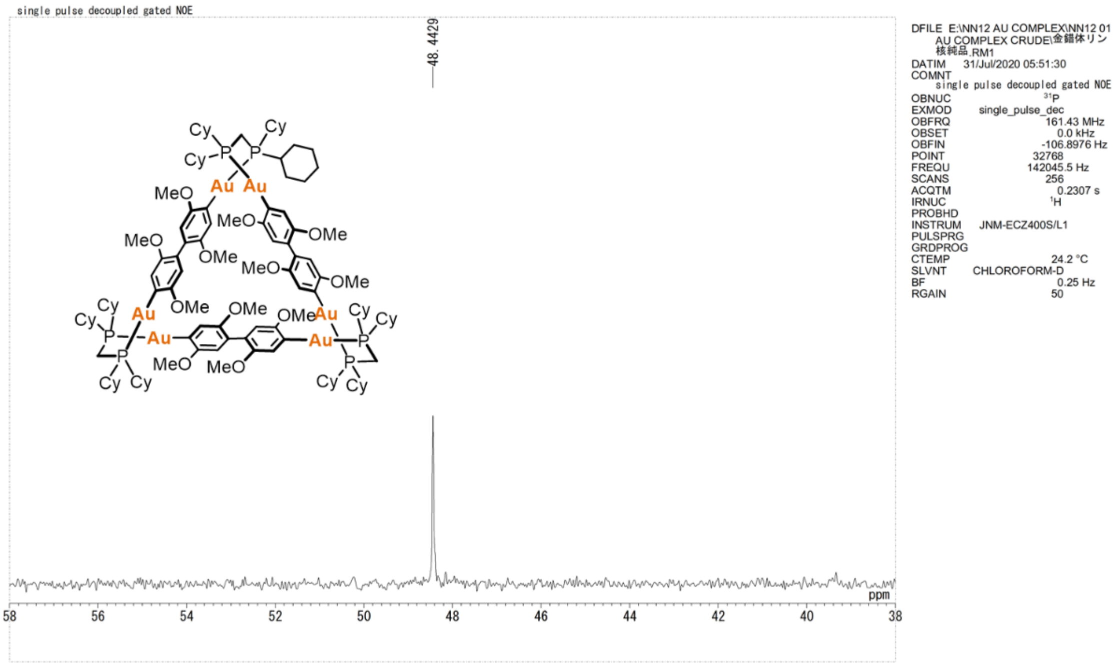

Supplementary Fig. 3 $^{31}P\{^1H\}$ NMR spectrum of macrocyclic Au complex, $[Au_2(C_6H_4\text{-}2,5\text{-}OMe)_2(Cy_2PCH_2PCy_2)]_3$ (**Au-1**) (161 MHz, $CDCl_3$, 297 K).

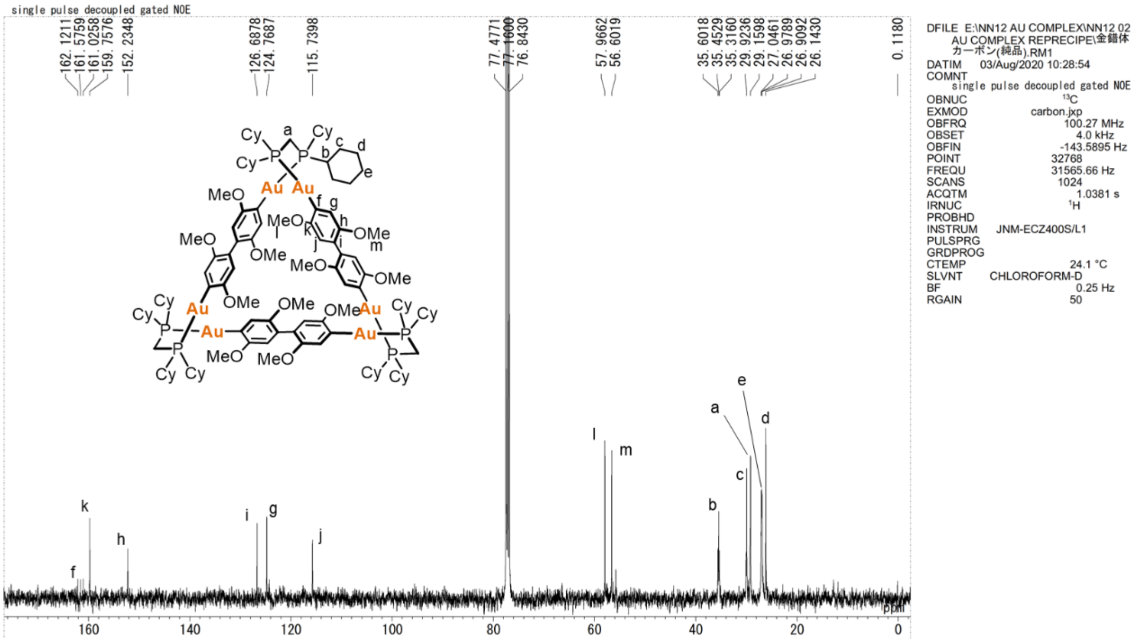

Supplementary Fig. 4 $^{13}C\{^{1}H\}$ NMR spectrum of macrocyclic Au complex, $[Au_2(C_6H_4\text{-}2,5\text{-}OMe)_2(Cy_2PCH_2PCy_2)]_3$ (**Au-1**) (100 MHz, $CDCl_3$, 297 K).

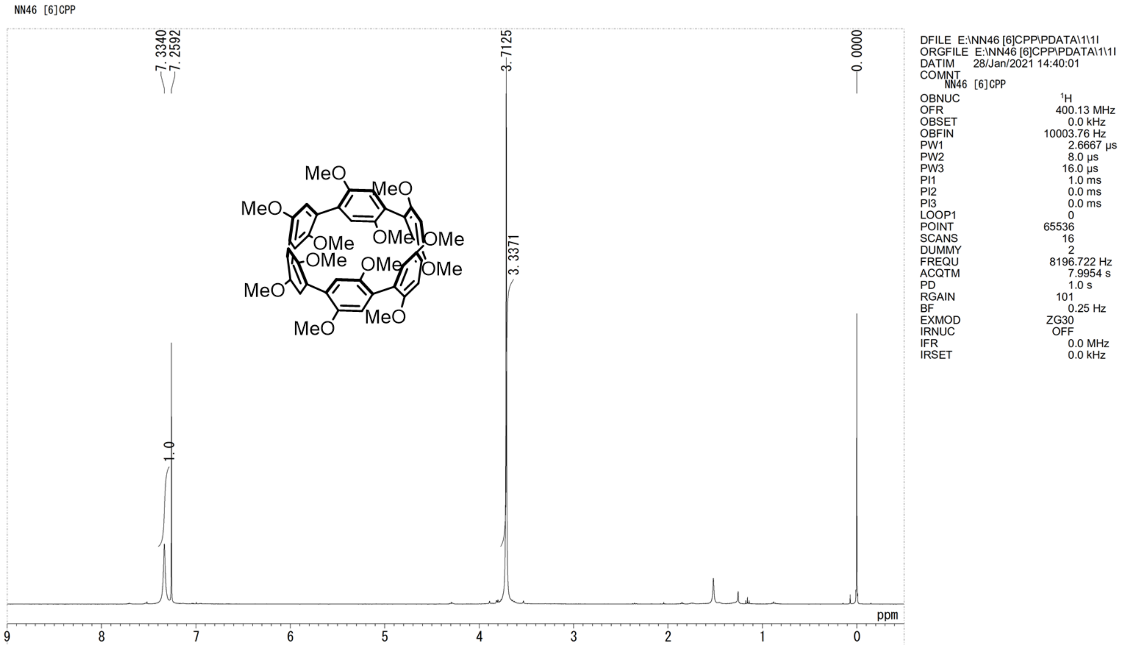

Supplementary Fig. 5 ^{1}H NMR spectrum of [6]CPP-12OMe (**1**) (400 MHz, $CDCl_3$, 297 K).

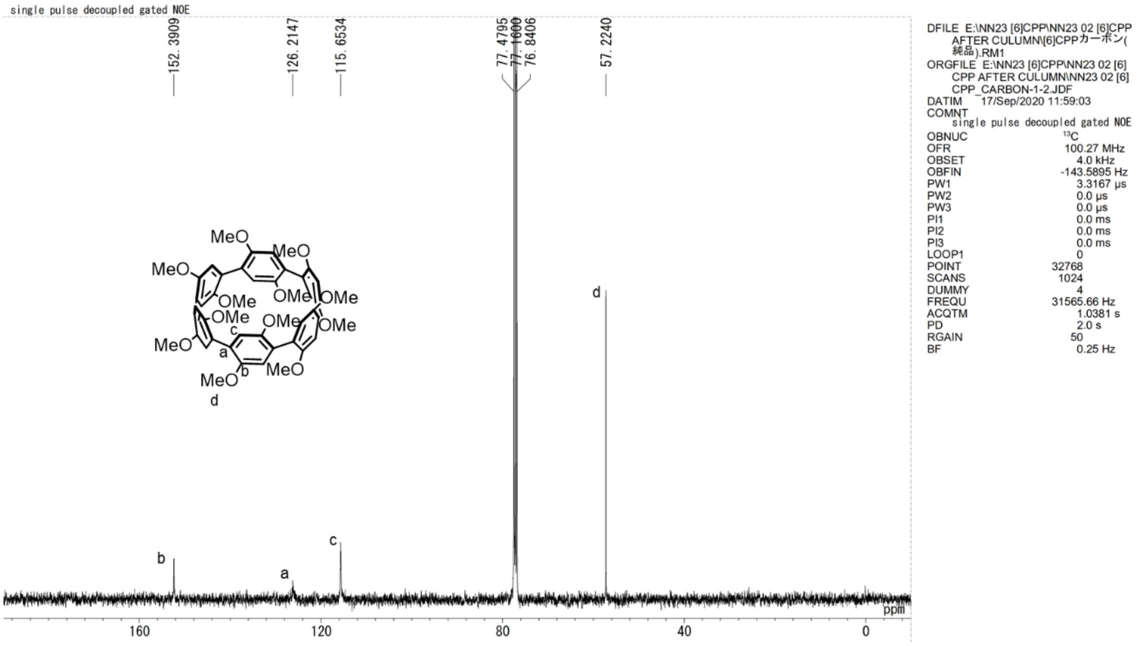

Supplementary Fig. 6 $^{13}C\{^1H\}$ NMR spectrum of [6]CPP-12OMe (**1**) (100 MHz, $CDCl_3$, 297 K).

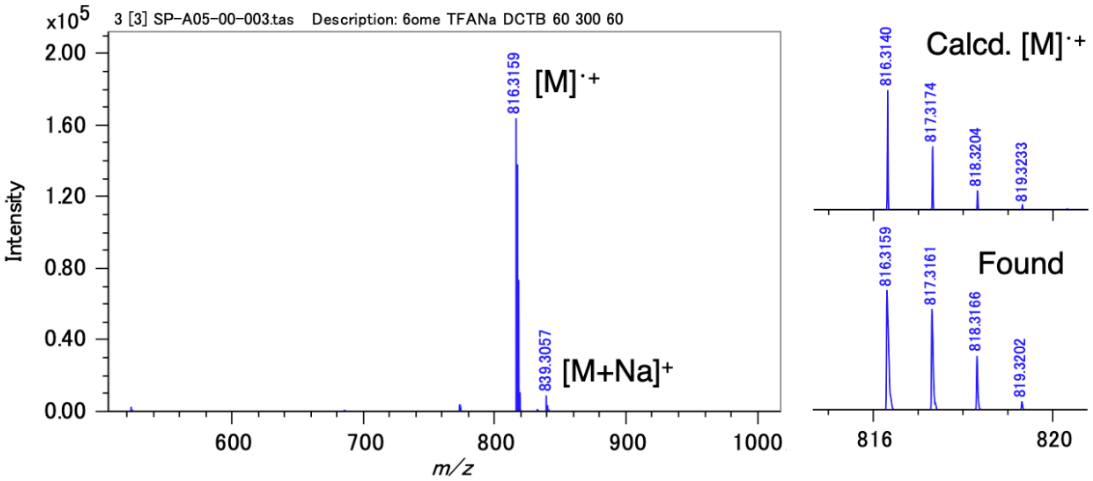

Supplementary Fig. 7 HRMS spectrum of [6]CPP-12OMe (**1**) (MALDI-TOF, DCTB; Ionizer, NaTFA).

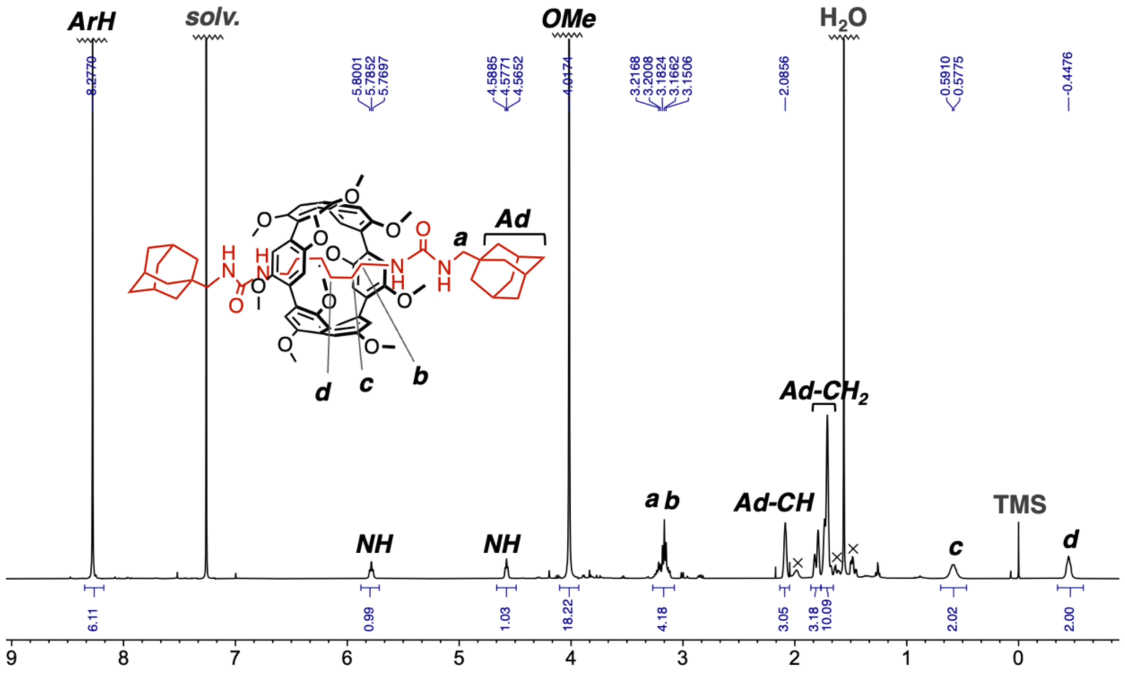

Supplementary Fig. 8 ^{1}H NMR spectrum of [2]rotaxane (**2**) (400 MHz, $CDCl_3$, 297 K).

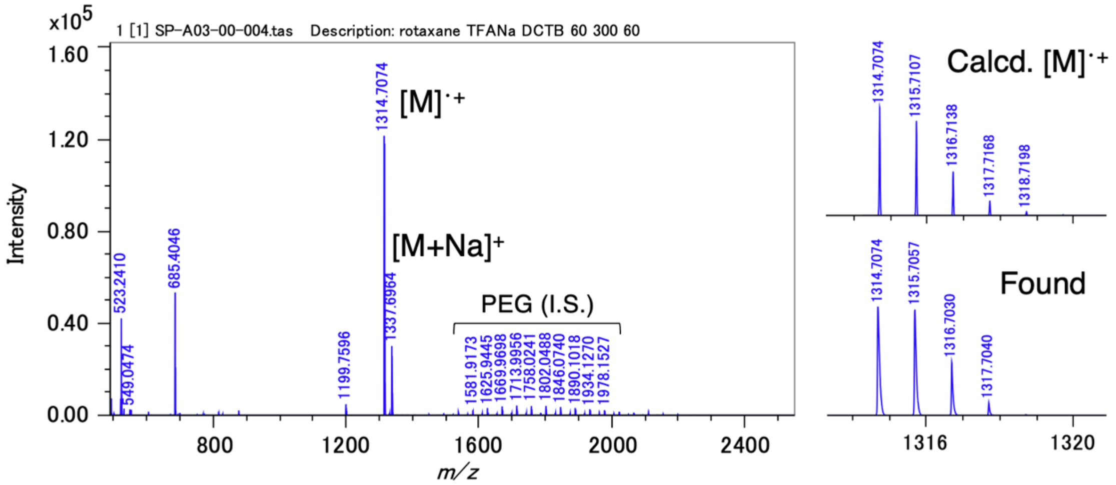

Supplementary Fig. 9 HRMS spectrum of [2]rotaxane (**2**) (MALDI-TOF, DCTB; Ionizer, NaTFA).

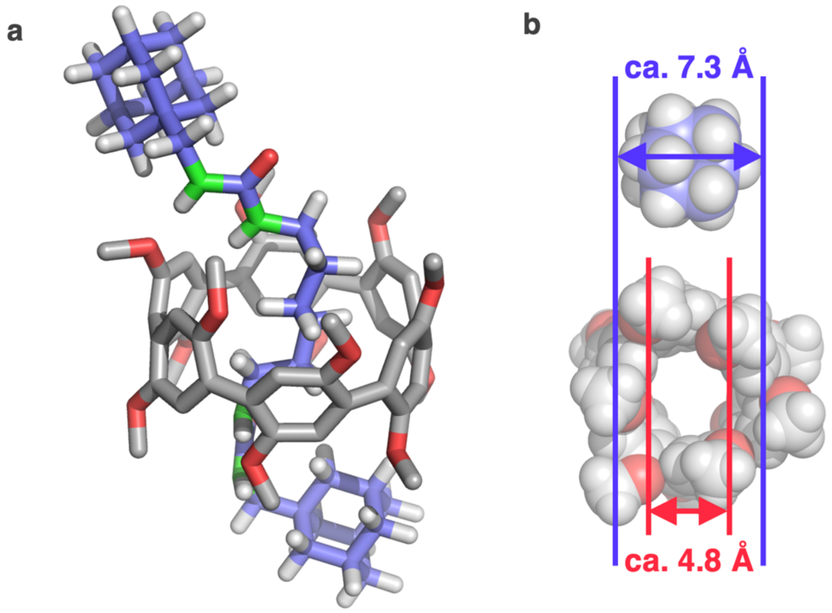

Supplementary Fig. 10 Optimized structure of [2]rotaxane (**2**) by DFT calculation. (a) Overall view with stick representation. Hydrogen atoms of CPP unit were omitted for clarity. (b) Size comparison of CPP unit and axle unit with CPK representation. The molecular size of the end-capping group was determined by adding the sum of the van der Waals radii of the two hydrogen atoms (1.2 Å × 2) to the average distance between the hydrogen atoms at both ends in the adamantane group (4.9 Å). The cavity size in [6]CPP-12OMe (**1**) was determined by subtracting the sum of the van der Waals radii of the two carbon atoms (1.7 Å × 2) from the average diameter of **1** (8.2 Å). The size of the end-capping group (ca. 7.3 Å) is sufficiently larger than the size of the cavity in **1** (ca. 4.8 Å) suggesting that dethreading does not occur and the interlocking structure would be maintained, which is also supported by the MD calculations (Supplementary Movie 1).

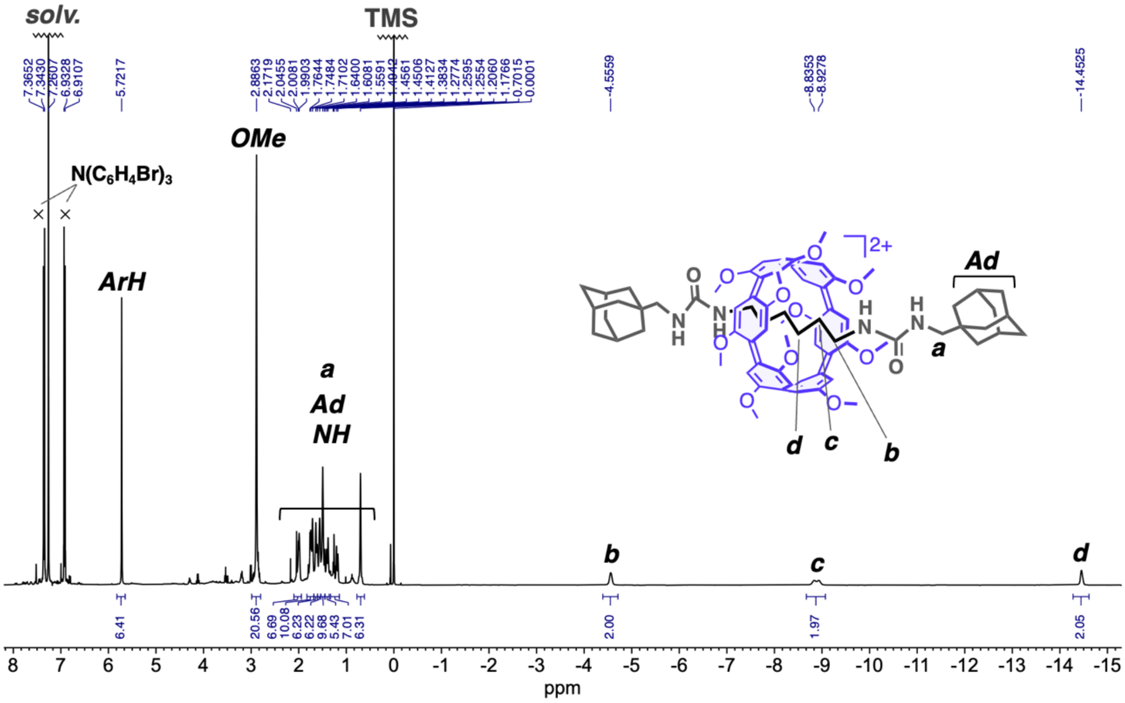

Supplementary Fig. 11 ^{1}H NMR spectrum of [2]rotaxane (**2**) upon addition of 2 equiv. of Magic Blue (400 MHz, $CDCl_3$, 297 K).

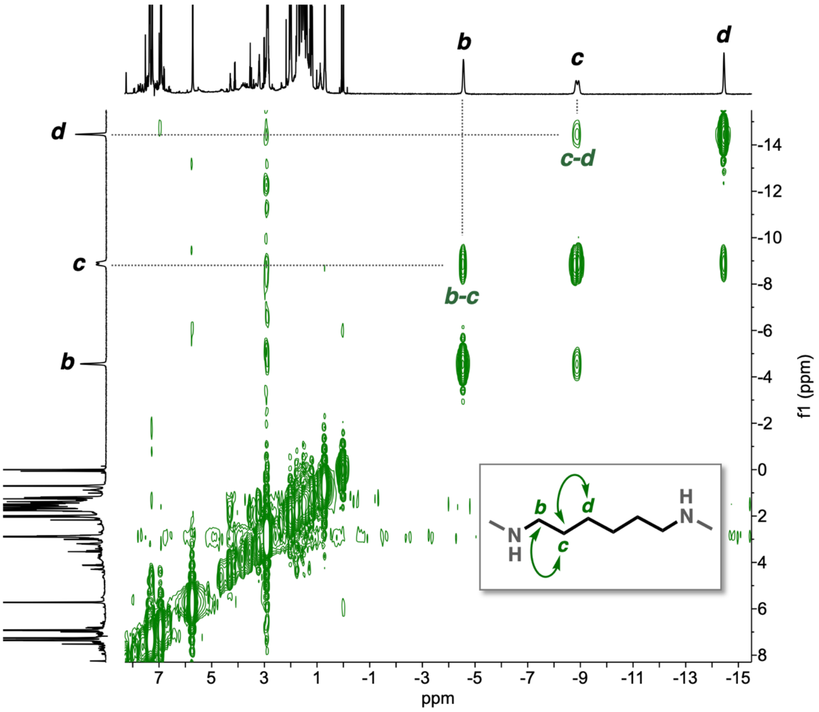

Supplementary Fig. 12 ^{1}H-^{1}H COSY spectrum of [2]rotaxane (**2**) upon addition of 2 equiv. of Magic Blue (400 MHz, $CDCl_3$, 297 K).

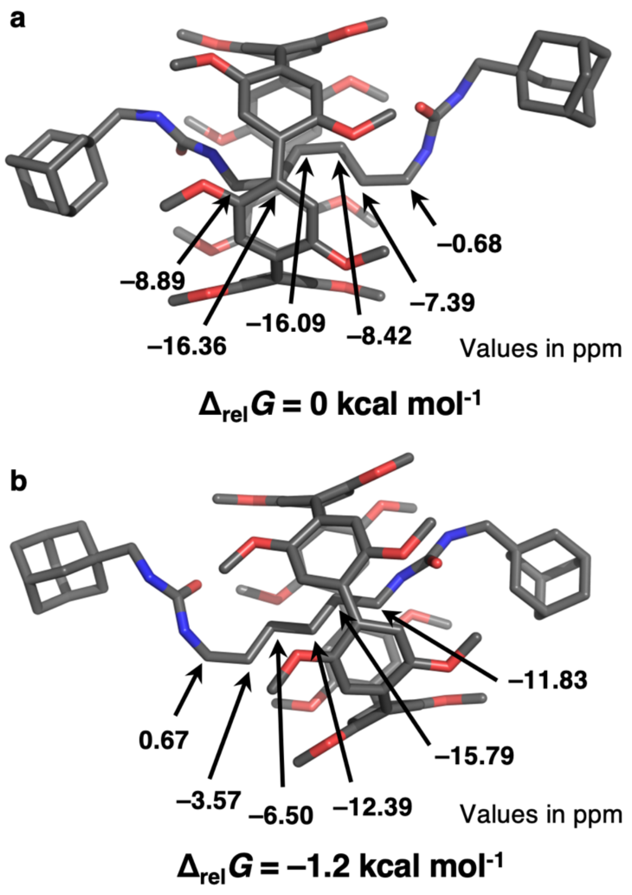

Supplementary Fig. 13 Optimized structures of [2]rotaxane-dication (**2**$^{2+}$) with different alkyl chain configuration; (a) gauche conformation, (b) anti conformation (hydrogen atoms were omitted. black, carbon; red, oxygen; blue, nitrogen; green). Values in the figure represent predicted gas-phase ^{1}H NMR chemical shifts (in ppm, tetramethylsilane (TMS) as the reference) of each proton of the alkyl chain (conformation search: CREST (iMTD-sMTD, GFN-FF), structure optimization: TPSS-D3(BJ)/def2-SVP, ORCA 4.2.1, NMR prediction: PBE0/pcSseg-1, ORCA 5.0.3, energy calculation: ωB97M-V/def2-TZVP, ORCA 5.0.3).

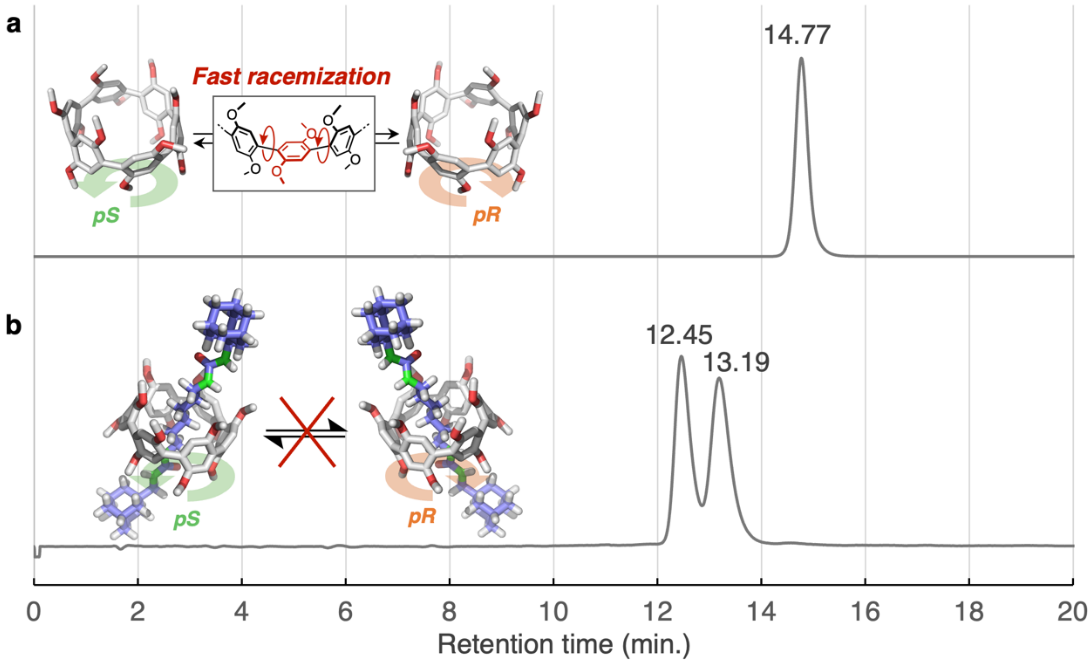

Supplementary Fig. 14 Chiral HPLC charts of (a) [6]CPP-12OMe (**1**) and (b) [2]rotaxane (**2**) (eluent: *n*-hexane/THF/*i*-PrOH = 70:25:5, CHIRALPAK® IA, r.t.).

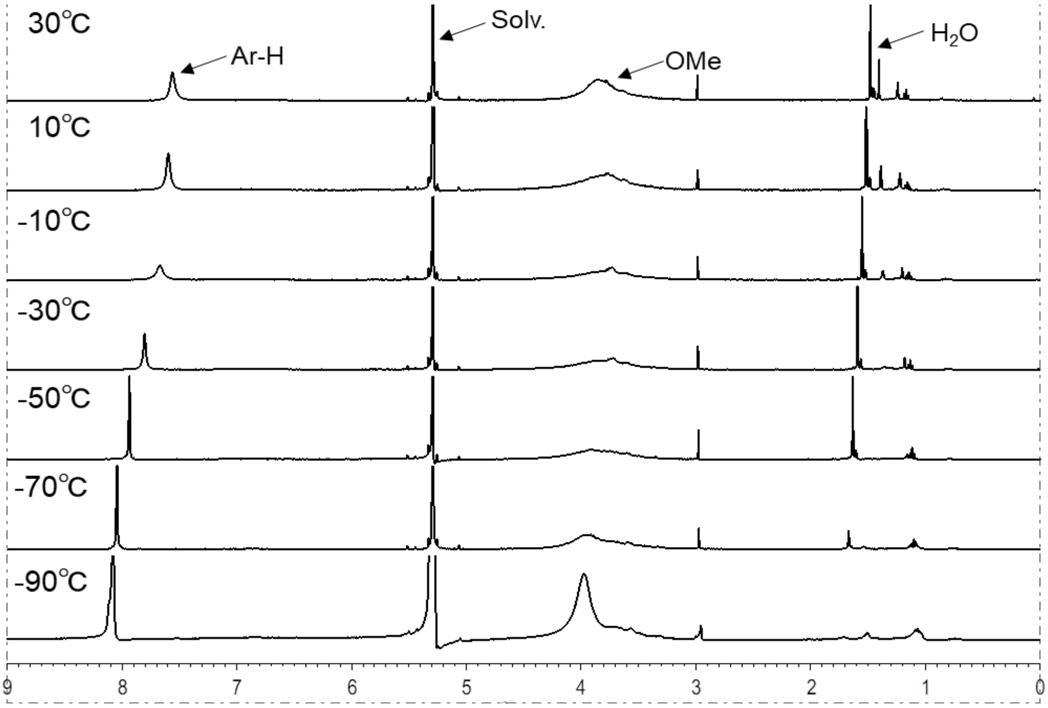

Supplementary Fig. 15 Variable-temperature ^{1}H NMR spectra of [6]CPP-12OMe (**1**) (400 MHz, CD_2Cl_2).

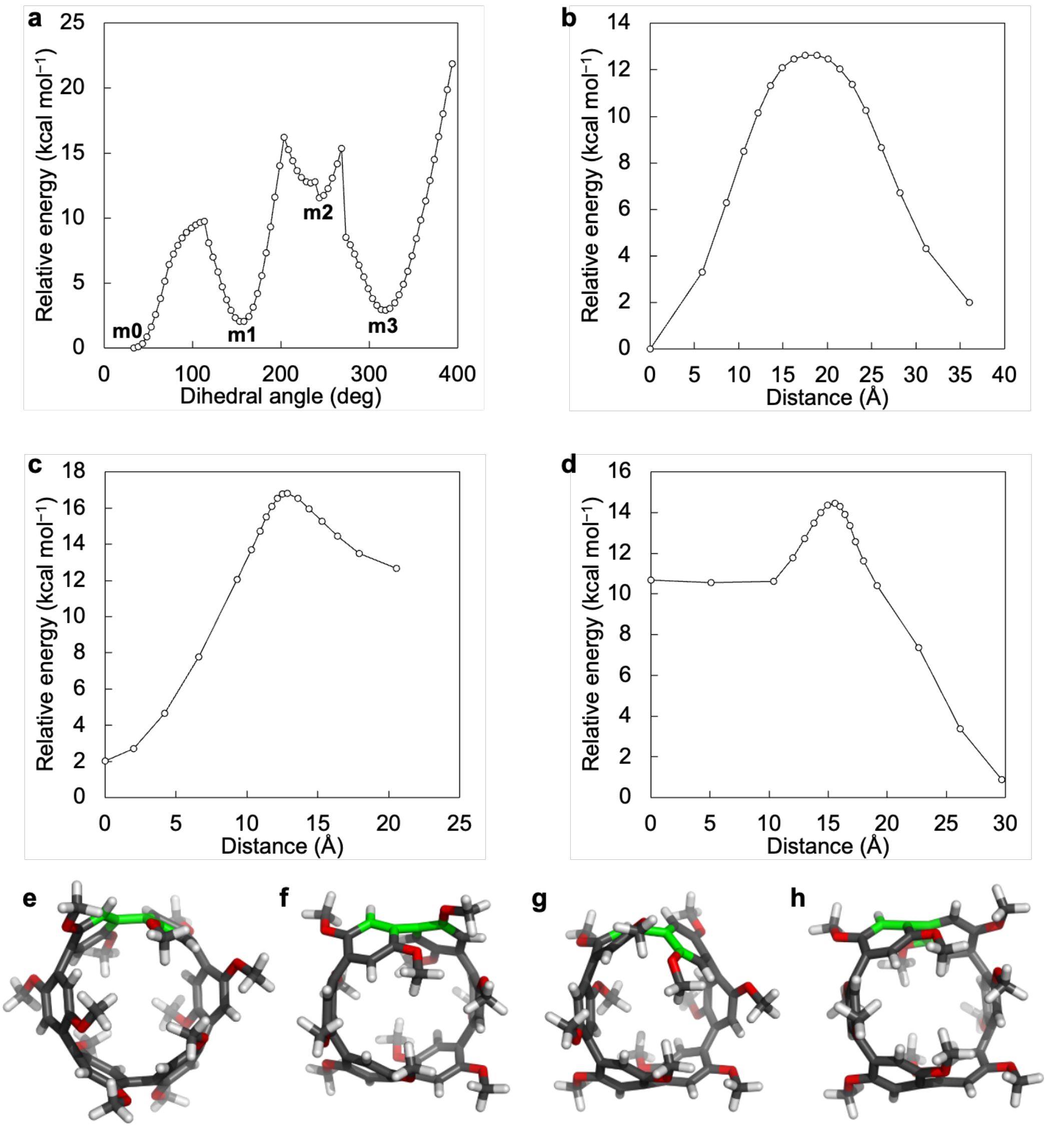

Supplementary Fig. 16 Phenylene flipping behavior of [6]CPP-12OMe (**1**). (a) Relaxed potential energy curve of dihedral angle between the neighboring phenylene units. (b) Result of climbing image nudged elastic band calculation (CI-NEB) calculation from the **m0** (fully optimized minima) to re-optimized **m1**. (c) Result of CI-NEB calculation from the re-optimized **m1** to re-optimized **m2** corresponding rate-determining step due to repulsion of methoxy group ($\Delta^{\ddagger}E$ = 16.8 kcal mol^{-1}). (d) Result of CI-NEB calculation from the re-optimized **m2** to re-optimized **m3**. (e) Geometry of **m0**. (f) Geometry of re-optimized **m1**. (g) Geometry of re-optimized **m2**. (h) Geometry of re-optimized **m3**. Relative energies in (a)–(d) referenced to the energy of **m0**. Green color in (e)–(h) denotes atoms scanned dihedral angle.

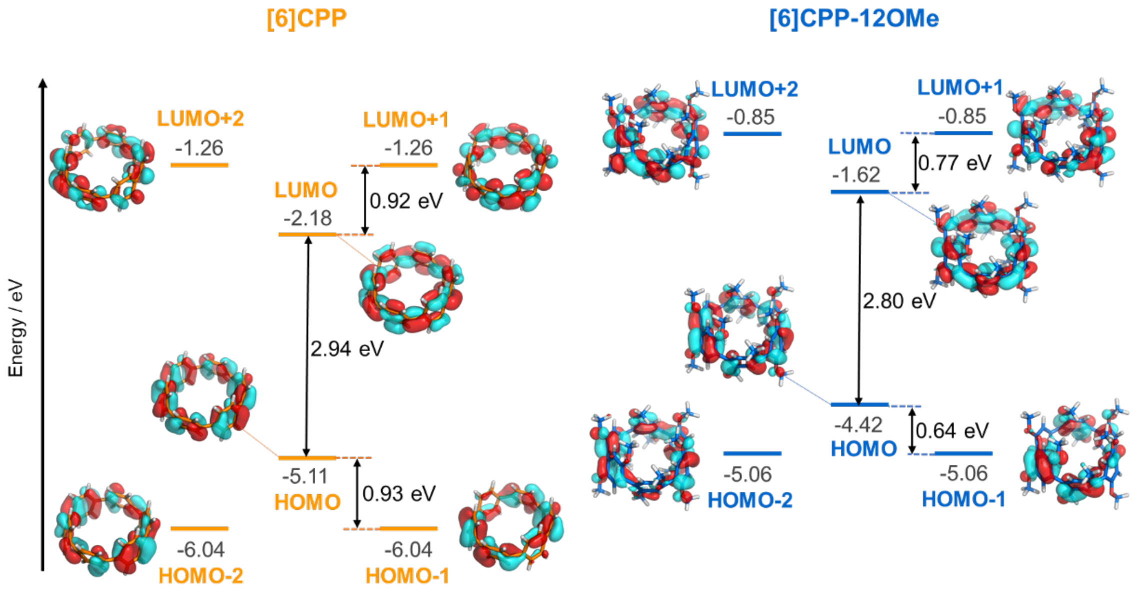

Supplementary Fig. 17 Energy diagrams and Kohn-Sham orbitals of [6]CPP (left) and [6]CPP-12OMe (**1**) (right) (B3LYP/def2-SVP//TPSS-D3(BJ)/def2-SVP).

Supplementary Table 1 Orbital energies of [6]CPP (B3LYP/def2-SVP//TPSS-D3(BJ)/def2-SVP).

MO	Occupation	Energy (eV)	ΔE_{MO} (eV)
117	2	-6.04	-
118	2	-6.04	0.93
119 (HOMO)	2	-5.11	2.94
120 (LUMO)	0	-2.18	0.92
121	0	-1.26	-
122	0	-1.26	-

Supplementary Table 2 Orbital energies of [6]CPP-12OMe (**1**) (B3LYP/def2-SVP//TPSS-D3(BJ)/def2-SVP).

MO	Occupation	Energy (eV)	ΔE_{MO} (eV)
213	2	-5.06	-
214	2	-5.06	0.64
215 (HOMO)	2	-4.42	2.80
216 (LUMO)	0	-1.62	0.77
217	0	-0.85	-
218	0	-0.85	-

Supplementary Table 3 Strain energies of [6]CPP and [6]CPP-12OMe (**1**) simulated by homodesmotic reactions[1] (ωB97M-V/def2-TZVP//TPSS-D3(BJ)/def2-SVP).

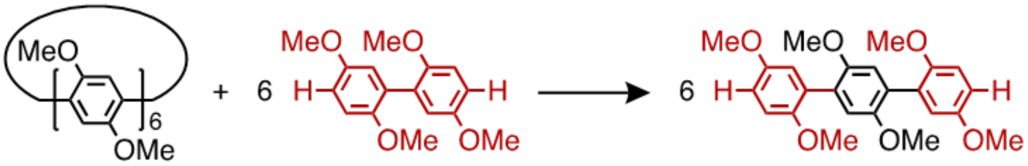

Entry	Strain energy (kcal mol^{-1})
[6]CPP	92.6
[6]CPP-12OMe (**1**)	88.2

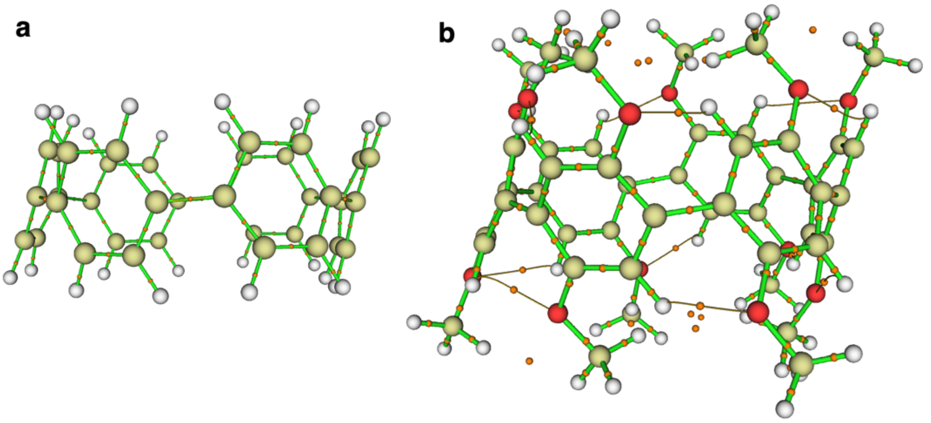

Supplementary Fig. 18 Bond critical points (BCPs) and bond path analysis based on Quantum theory of atoms in molecules (QTAIM) of (a) [6]CPP and (b) [6]CPP-12OMe (**1**) (Wavefunction: ORCA 4.2.1, TPSS-D3(BJ)/def2-SVP, QTAIM: Multiwfn). The bond paths regarding the hydrogen bonds and bond critical points (gray lines and orange dots, respectively) are found between aryl hydrogens and methoxy oxygens in **1**, indicating the existence of hydrogen bonds.

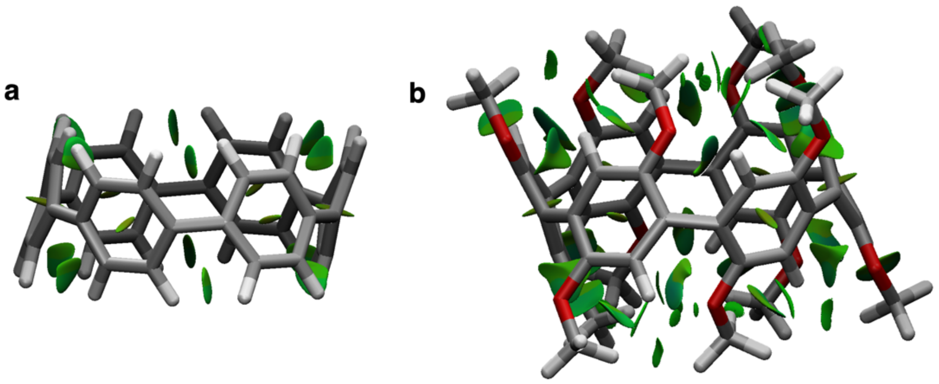

Supplementary Fig. 19 Non-Covalent Interaction (NCI) plots of (a) [6]CPP and (b) [6]CPP-12OMe (**1**). The reduced density gradient (RDG) isosurfaces (isovalue = 0.3 a.u.) are visualized as green surfaces (Wavefunction: ORCA 4.2.1, TPSS-D3(BJ)/def2-SVP, NCI: NCIPLOT).

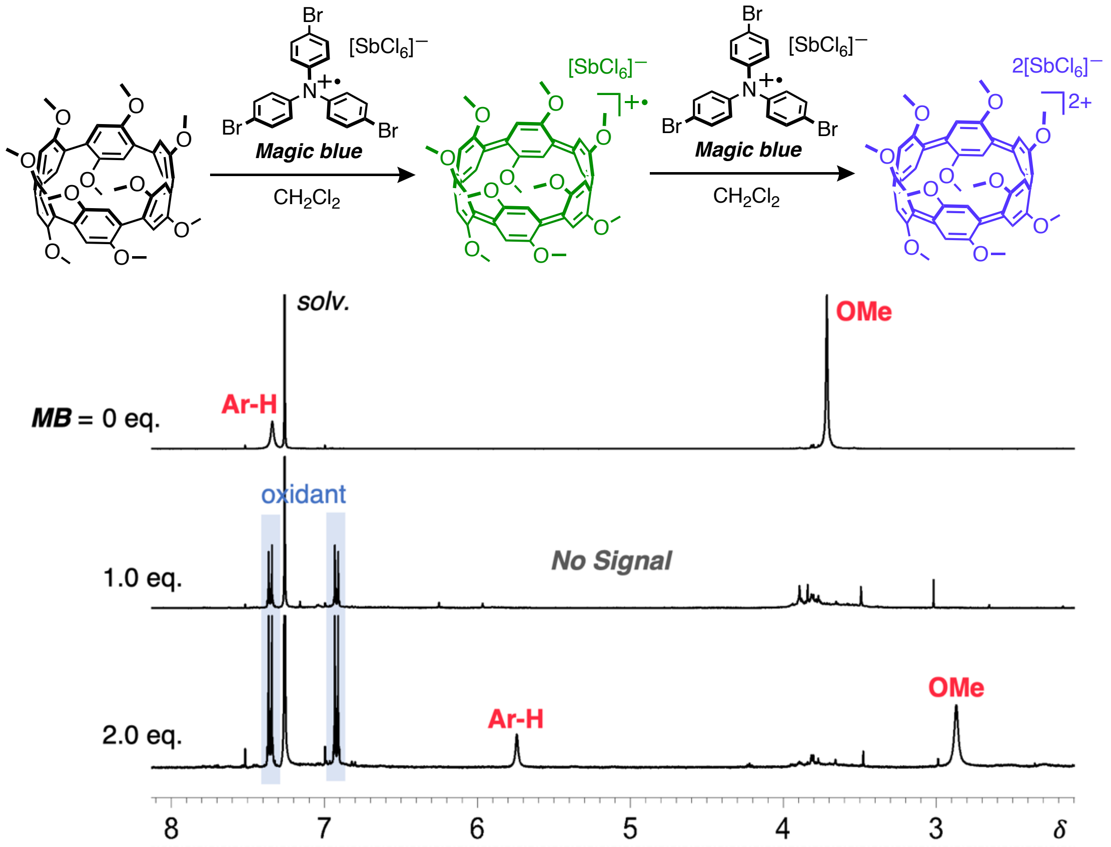

Supplementary Fig. 20 ^{1}H NMR titration of [6]CPP-12OMe (**1**) with Magic Blue, **MB** (400 MHz, $CDCl_3$, 297 K).

Supplementary Table 4 The first five states of calculated transition properties and frontier orbitals (SCF level) of neutral [6]CPP-12OMe (SOS-ωPBEPP86/def2-SVP//TPSS-D3(BJ)/def2-SVP).

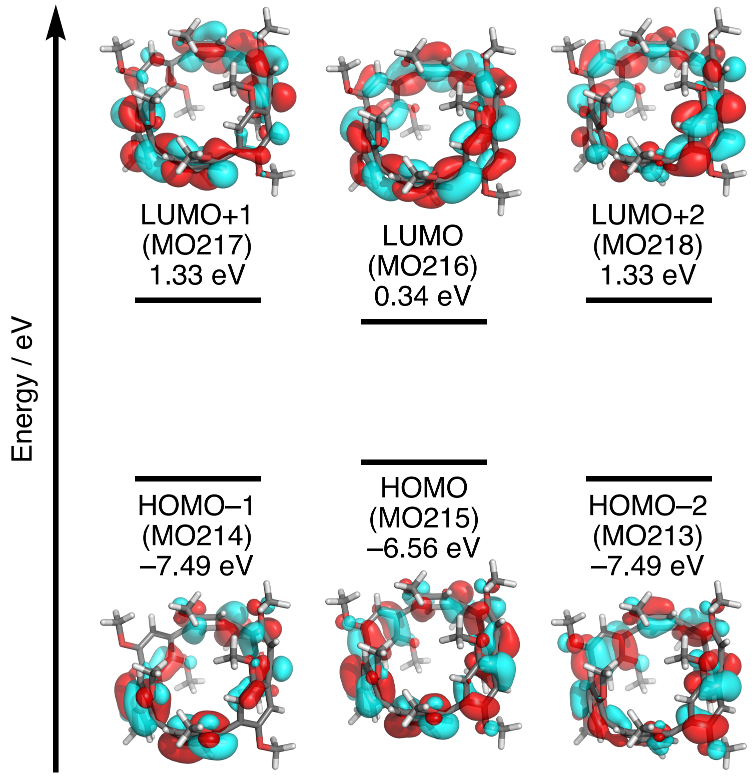

State	Wavelength (nm)	f_{osc}	Assign (above 10%)
1	431	0.0129	HOMO→LUMO, 77%
2	338	0.845	HOMO–1→LUMO, 44%<br>HOMO→LUMO+1, 38%
3	338	0.845	HOMO–2→LUMO, 44%<br>HOMO→LUMO+2, 38%
4	316	0.153	HOMO–4→LUMO, 24%<br>HOMO–3→LUMO, 15%
5	316	0.153	HOMO–3→LUMO, 24%<br>HOMO–4→LUMO, 15%

Supplementary Table 5 The first five states of calculated transition properties and frontier orbitals (SCF level) of [6]CPP-12OMe cation radical (SOS-ωPBEPP86/def2-SVP//TPSS-D3(BJ)/def2-SVP).

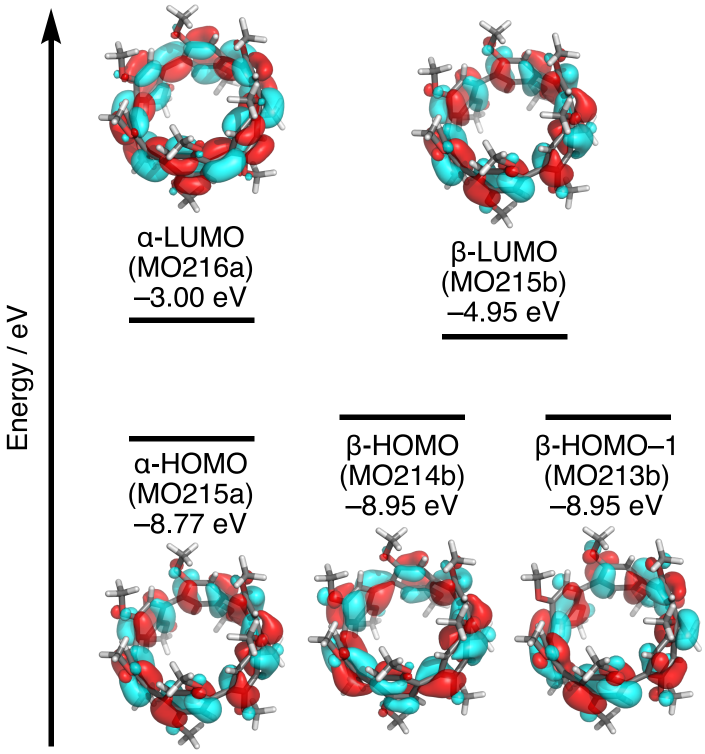

State	Wavelength (nm)	f_{osc}	Assign (above 10%)
1	1334	0.165	β-HOMO→β-LUMO, 85%
2	1331	0.165	β-HOMO–1→β-LUMO, 85%
3	825	0.00421	α-HOMO→α-LUMO, 70%
4	631	0.0212	β-HOMO→β-LUMO+1, 43%<br>α-HOMO–1→α-LUMO, 18%<br>α-HOMO→α-LUMO+1, 18%
5	630	0.0209	β-HOMO–1→β-LUMO+1, 43%<br>α-HOMO–2→α-LUMO, 18%<br>α-HOMO→α-LUMO+2, 18%

Supplementary Table 6 The first five states of calculated transition properties and frontier orbitals (SCF level) of [6]CPP-12OMe dication (SOS-ωPBEPP86/def2-SVP//TPSS-D3(BJ)/def2-SVP).

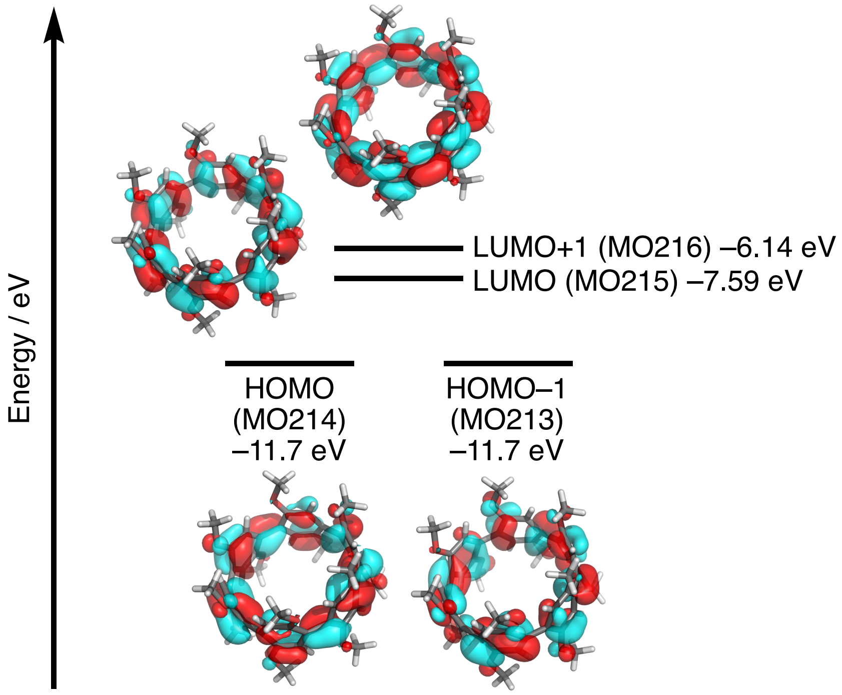

State	Wavelength (nm)	f_{osc}	Assign (above 10%)
1	971	0.214	HOMO→LUMO, 69%<br>HOMO–1→LUMO+1, 27%
2	970	0.214	HOMO–1→LUMO, 69%<br>HOMO→LUMO+1, 27%
3	680	2.1×10^{8}	HOMO–2→LUMO, 94%
4	680	4.9×10^{8}	HOMO–3→LUMO, 94%
5	653	5.6×10^{8}	HOMO–4→LUMO, 95%

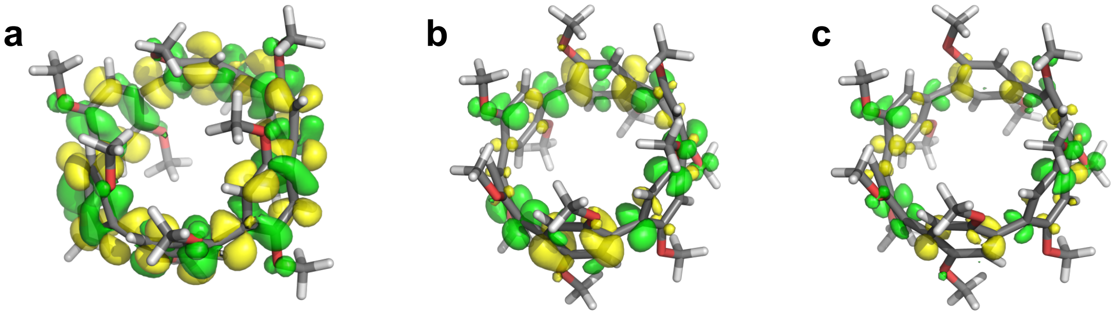

Supplementary Fig. 21 Density differences of the first excitation state of (a) neutral, (b) cation radical, and (c) dication species of [6]CPP-12OMe. Yellow and green color corresponds to positive and negative values of the density difference, respectively (isosurface = 0.0005).

Supplementary Table 7 List of averaged NICS (nucleus-independent chemical shifts) values of [6]CPP-12OMe (**1**), cation radical (**1**$^{+\bullet}$), dication (**1**$^{2+}$), and [6]CPP dication [2].

	**1**	Cation radical (**1**$^{+\bullet}$)	Dication (**1**$^{2+}$)	[6]CPP dication [2]
NICS(0)	-9.7	-8.4	-10.0	
NICS(1), outside	-8.8	-7.0	-5.2	-5.5
NICS(1), inside	-10.8	-13.5	-22.8	-29.6

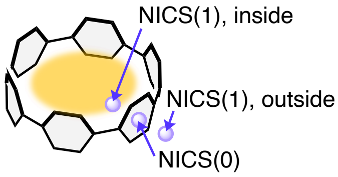

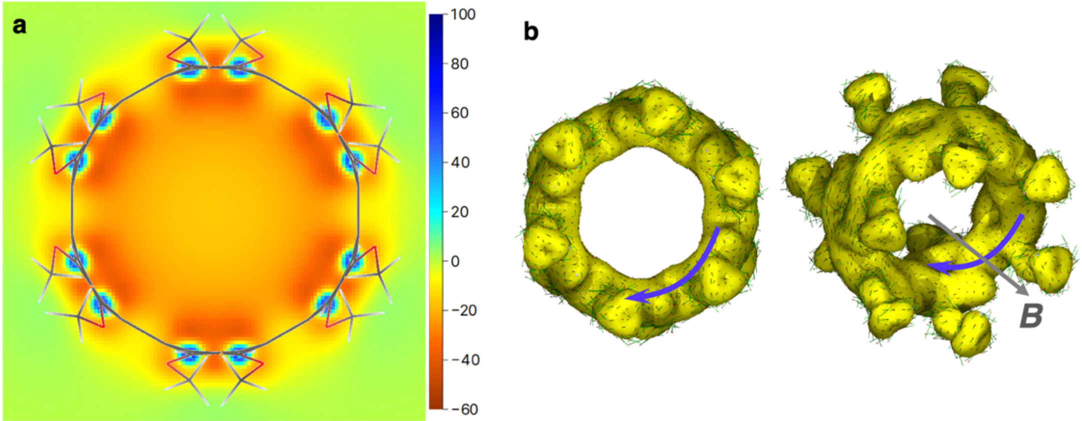

Supplementary Fig. 22 (a) 2D-NICS plot of dication (**1**$^{2+}$) (GIAO-PBE0/pcSseg-1//TPSS-D3(BJ)/def2-SVP), (b) anisotropy of the induced current density (ACID) plot of dication (**1**$^{2+}$) (CSGT-PBE0/pcSseg-1//TPSS-D3(BJ)/def2-SVP).

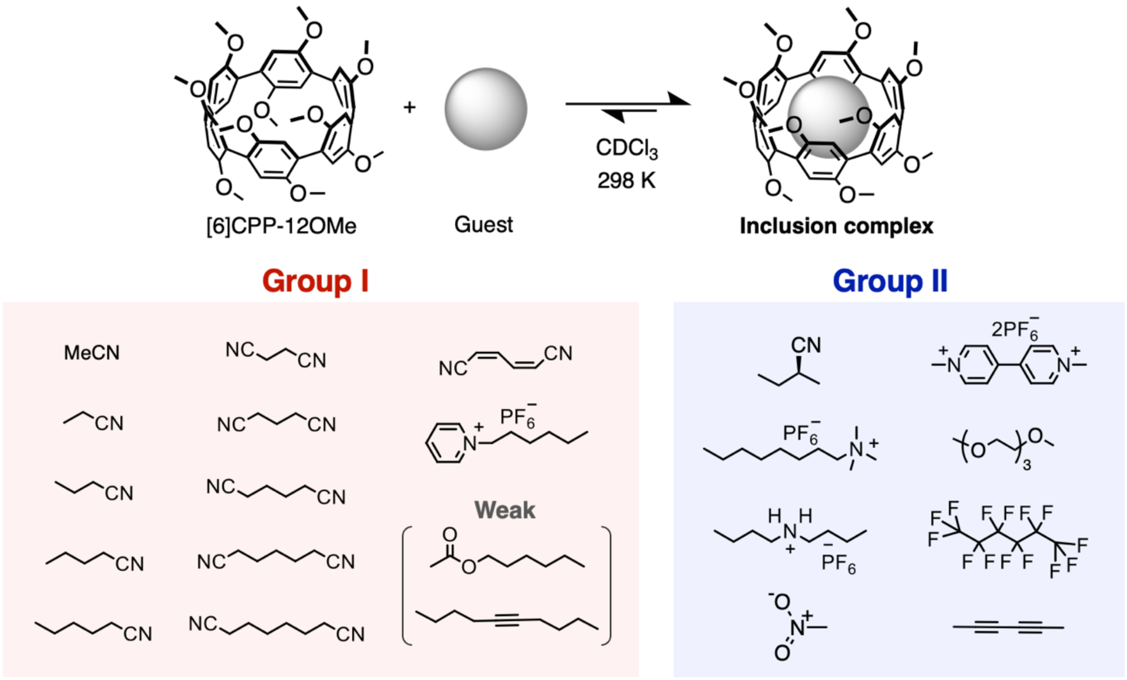

Supplementary Fig. 23 Results of guest inclusion test for [6]CPP-12OMe (**1**). Classification is based on whether the Ar-H chemical shift of ^{1}H NMR spectra changes (group I, Supplementary Figs. 24-25) or not (group II) upon addition of a large excess of the guest molecule to a $CDCl_3$ solution of **1** (Supplementary Fig. 26).

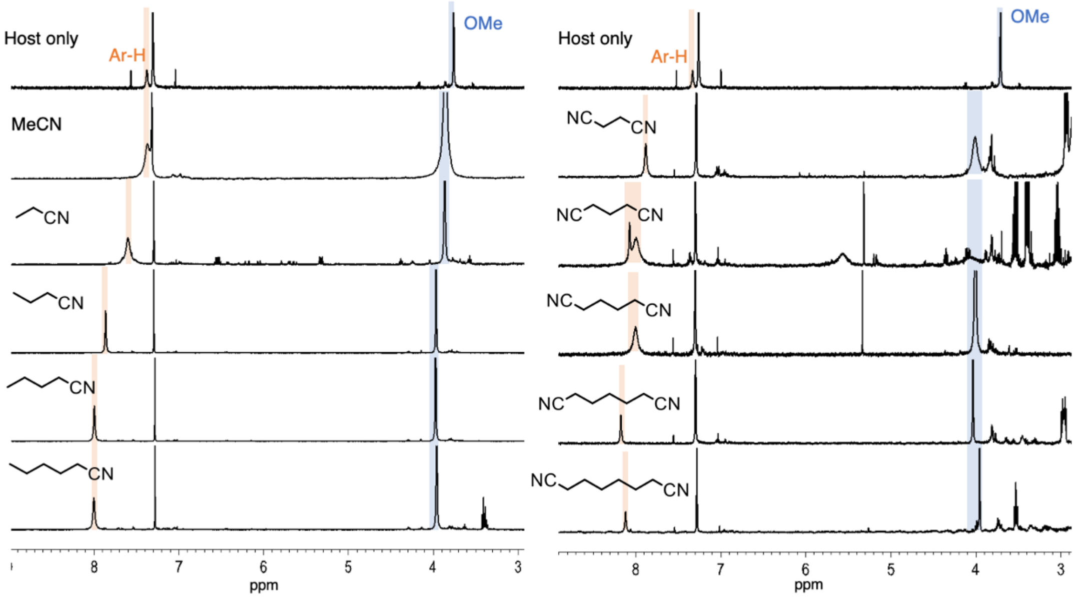

Supplementary Fig. 24 Stacked ^{1}H NMR spectra of [6]CPP-12OMe (**1**) with excess amount of cyanoalkanes (400 MHz, $CDCl_3$, 297 K).

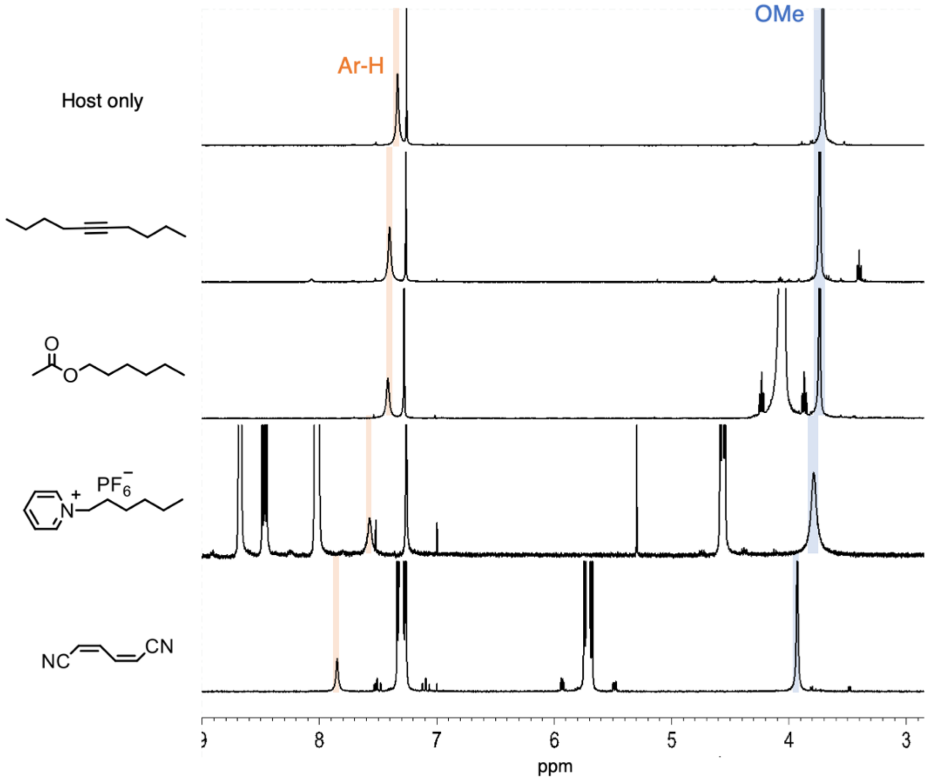

Supplementary Fig. 25 Stacked ^{1}H NMR spectra of [6]CPP-12OMe (**1**) with excess amount of various guest molecules (400 MHz, $CDCl_3$, 297 K).

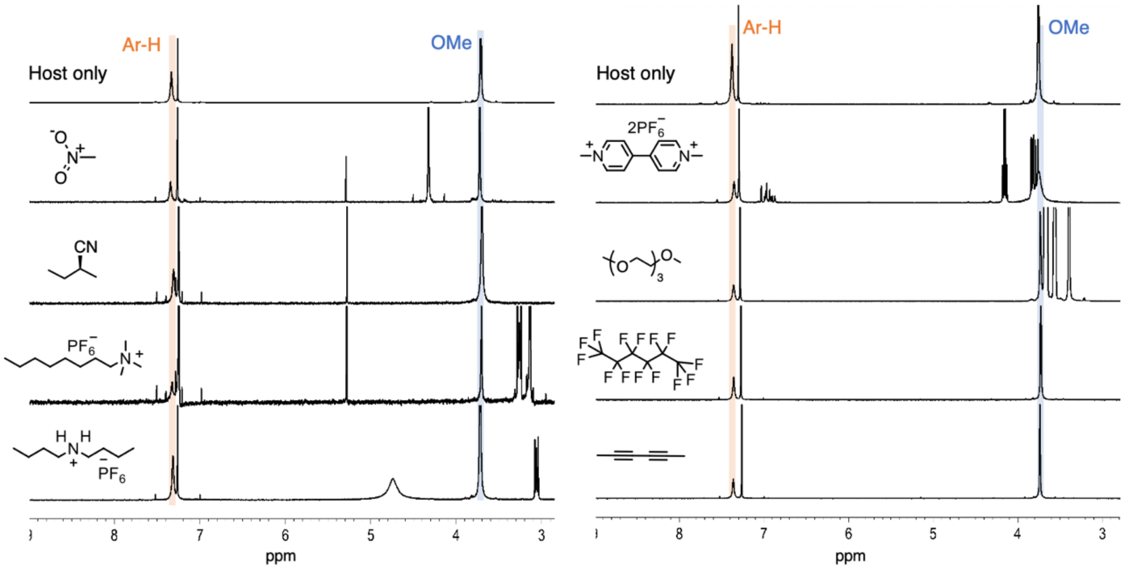

Supplementary Fig. 26 Stacked ^{1}H NMR spectra of [6]CPP-12OMe (**1**) with excess amount of bulky or electron-deficient guest molecules (400 MHz, $CDCl_3$, 297 K).

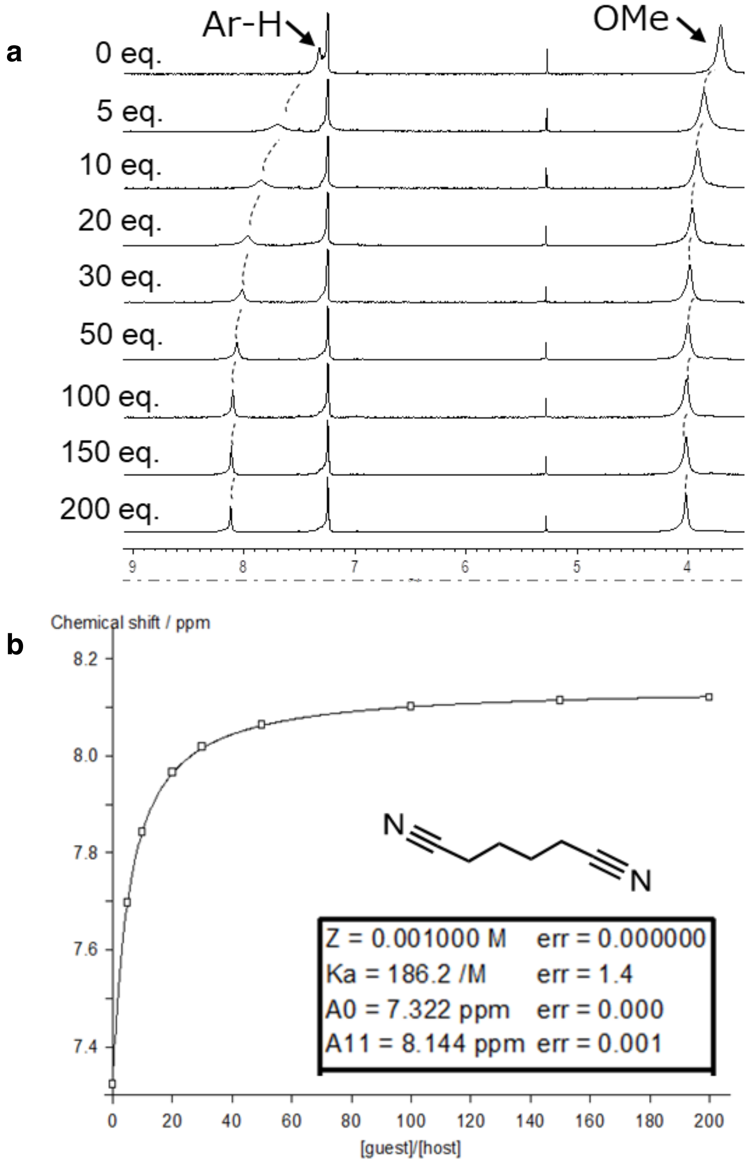

Supplementary Fig. 27 (a) ^{1}H NMR titration and (b) titration profile of Ar-H proton of [6]CPP-12OMe (**1**) with NC-$(CH_2)_4$-CN (adiponitrile, **1**) (400 MHz, $CDCl_3$, 297 K).

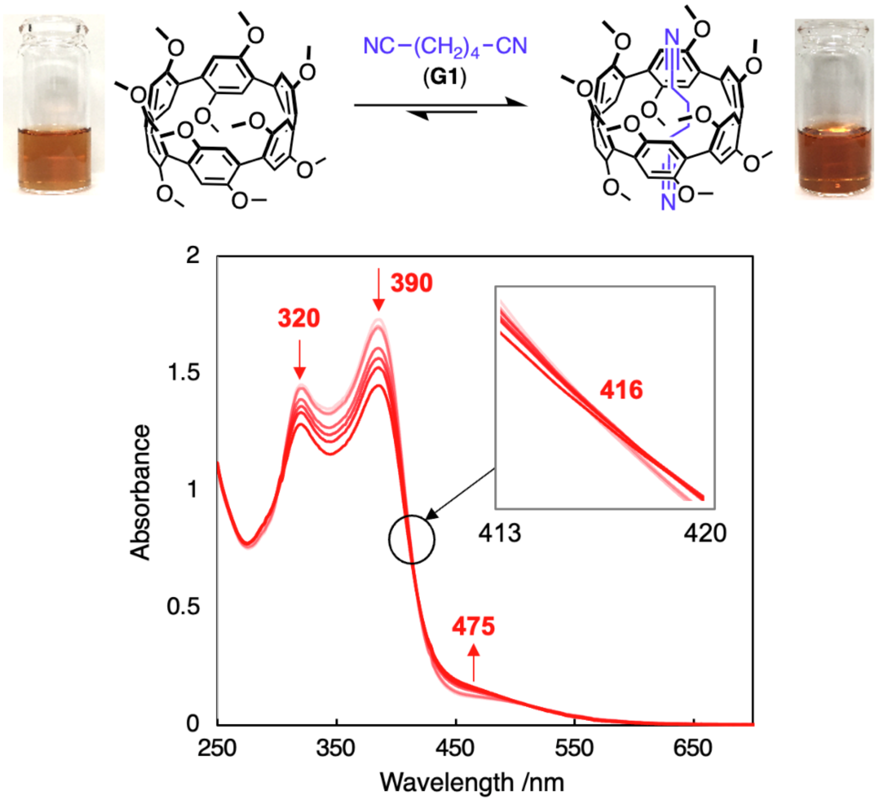

Supplementary Fig. 28 UV/vis absorption titration of [6]CPP-12OMe (**1**) with NC-(CH_2)$_4$-CN (adiponitrile, **G1**) (CH_2Cl_2, r.t., [**1**] = 0.050 mmol L^{-1}).

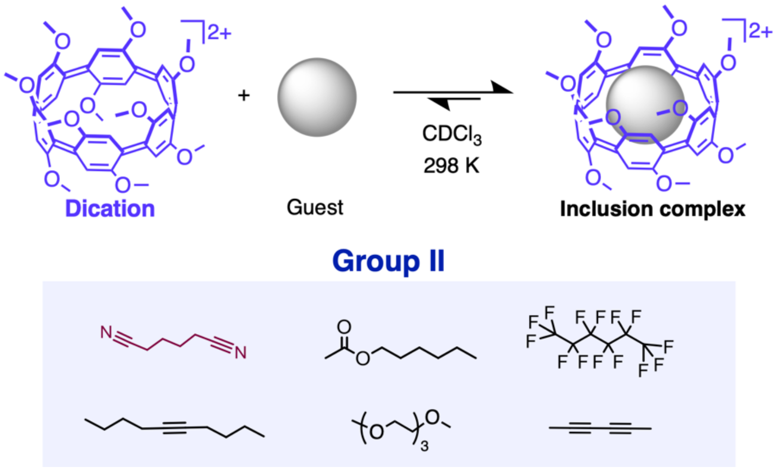

Supplementary Fig. 29 Results of guest inclusion test for [6]CPP-12OMe dication (1^{2+}). Classification is based on whether the Ar-H chemical shift of ^1H NMR spectra changes (group I, not applicable) or not (group II, Supplementary Fig. 30) upon addition of a large excess of the guest molecule to a $CDCl_3$ solution of 2^{2+}.

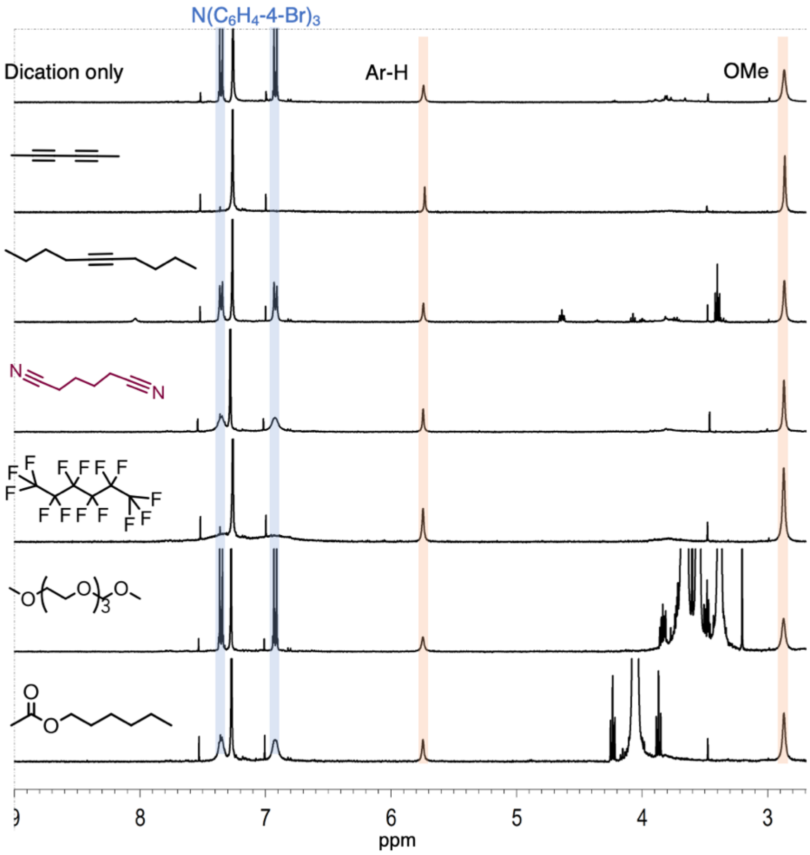

Supplementary Fig. 30 Stacked ^1H NMR spectra of [6]CPP-12OMe dication (1^{2+}) with excess amount of various guest molecules (400 MHz, $CDCl_3$, 297 K).

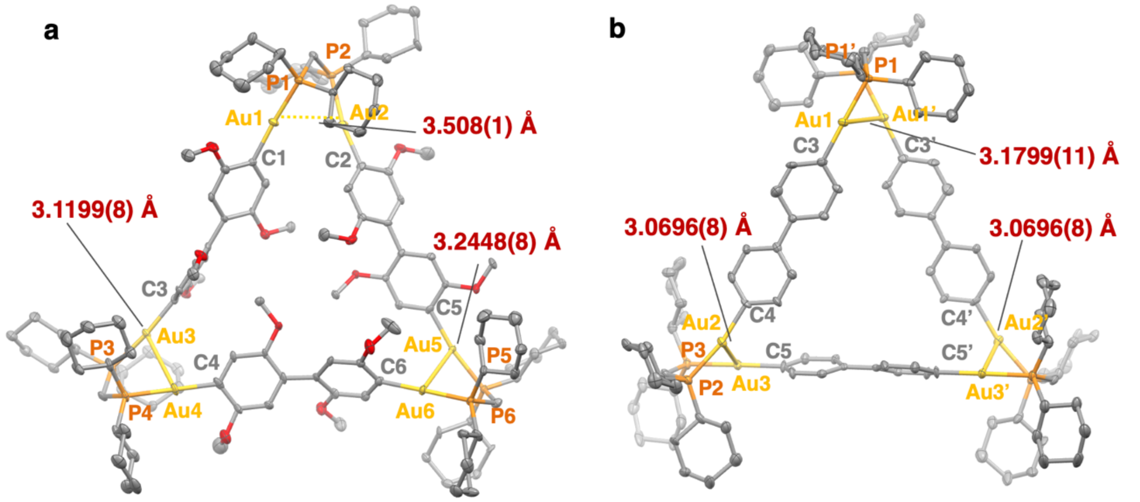

Supplementary Fig. 31 ORTEP drawings of macrocyclic Au complex (30% level of probability): (a) $[Au_2(C_6H_4\text{-}2,5\text{-}OMe)_2(Cy_2PCH_2PCy_2)]_3$ (**Au-1**), (b) $[Au_2(C_6H_4)_2(Cy_2PCH_2PCy_2)]_3$ (**Au-1’**) (our previous work [3], CCDC: 1994843).

Supplementary Table 8 List of selected atom distances and angles for **Au-1** and **Au-1’**[3].

	$d_{Au\text{-}Au}$ (Å)	$d_{Au\text{-}C}$ (Å)		$d_{Au\text{-}P}$ (Å)		Torsion angle (deg)
**Au-1**	3.508(1)<br>Au1-Au2	2.071(13)<br>Au1-C1	2.040(12)<br>Au4-C4	2.284(3)<br>Au1-P1	2.285(3)<br>Au4-P4	41.7(5)<br>C1-Au1-Au2-C2
	3.1199(7)<br>Au3-Au4	2.063(11)<br>Au2-C2	2.068(11)<br>Au5-C5	2.301(3)<br>Au2-P2	2.294(3)<br>Au5-P5	48.9(5)<br>C3-Au3-Au4-C4
	3.2448(8)<br>Au5-Au6	2.072(11)<br>Au3-C3	2.005(14)<br>Au6-C6	2.288(3)<br>Au3-P3	2.280(3)<br>Au6-P6	41.3(6)<br>C5-Au5-Au6-C6
**Au-1’**[3]	3.1799(11)<br>Au1-Au1’	2.04(1)<br>Au1-C3	2.05(1)<br>Au2-C5	2.288(3)<br>Au1-P1	2.295(4)<br>Au2-P2	58.5(6)<br>C3-Au1-Au1’-C3’
	3.0696(8)<br>Au2-Au3	2.10(1)<br>Au3-C5		2.298(3)<br>Au3-P3		48.7(6)<br>C4-Au2-Au3-C5

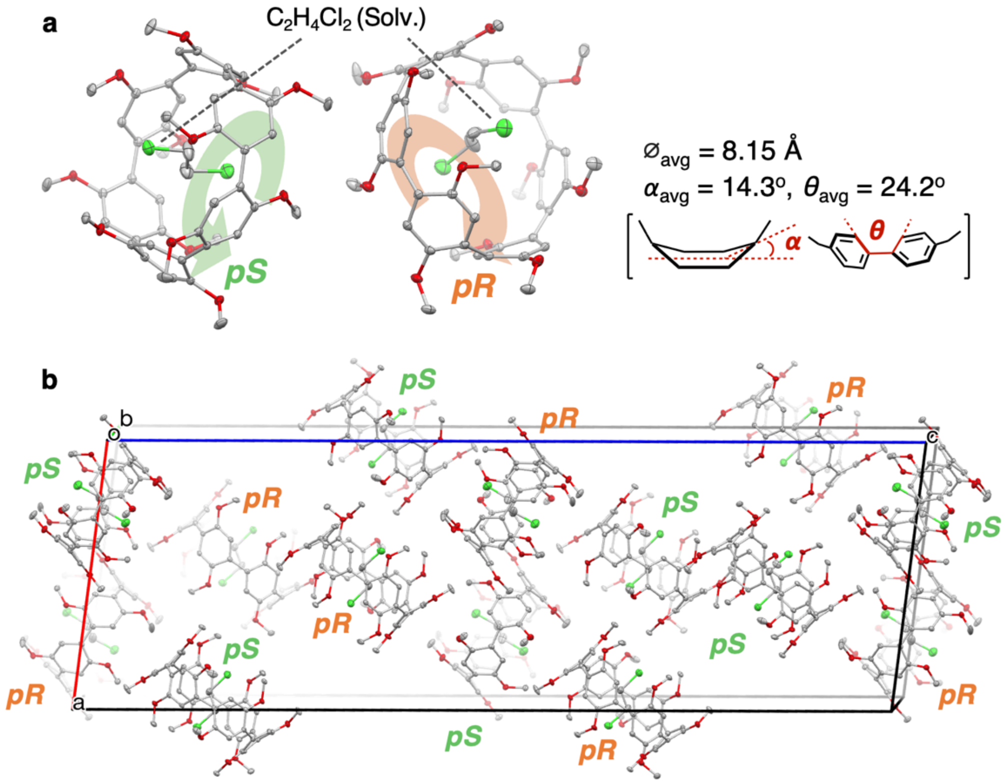

Supplementary Fig. 32 ORTEP drawing of [6]CPP-12OMe (**1**) (30% level of probability): (a) molecular structure (two isomers were analyzed as independent structures), (b) unit cell structure.

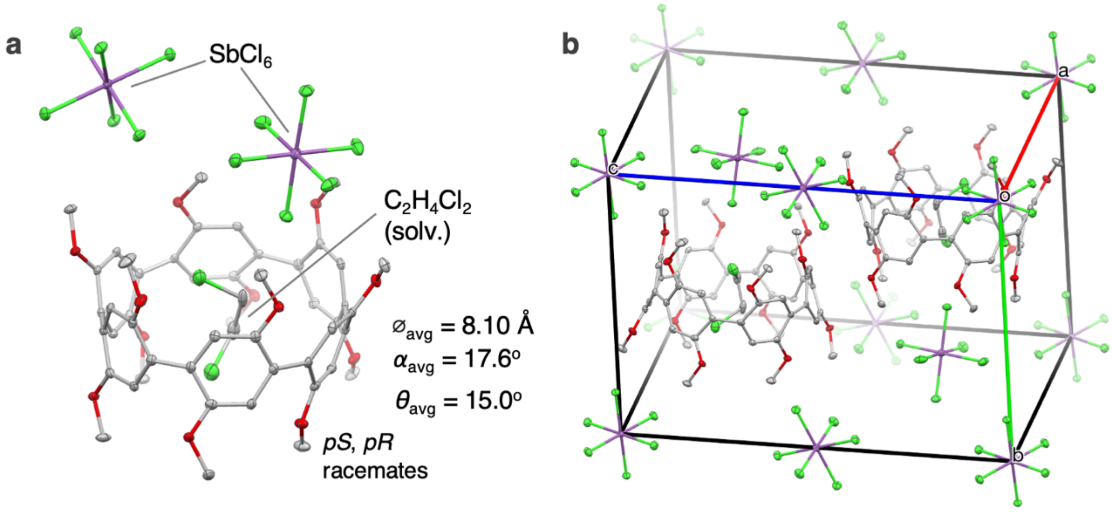

Supplementary Fig. 33 ORTEP drawing of [6]CPP-12OMe dication ($\mathbf{1}^{2+}$) (30% level of probability): (a) molecular structure (two isomers were analyzed as identical structure), (b) unit cell structure.

Supplementary Table 9 List of averaged bond length and diameters of [6]CPP-12OMe dication ($\mathbf{1}^{2+}$), [6]CPP-12OMe (**1**), [6]CPP, and 1,4-dimthoxybenzene.

Entry	*a* (Ar-Ar) (Å)	*b* (C-C-H) (Å)	*c* (Å)	*d* (C-C-O) (Å)	*e* (C-O) (Å)	⌀ (Å)
[6]CPP-12OMe dication ($\mathbf{1}^{2+}$)	1.4432	1.4227	1.3705	1.444	1.3546	7.946<br>7.965<br>8.375
[6]CPP-12OMe (**1**)	1.4925	1.4062	1.3857	1.4143	1.3758	8.084<br>8.085<br>8.283
[6]CPP [4]	1.4893	1.4038	1.3858	-	-	8.040<br>8.050<br>8.117
1,4-Dimthoxy benzene [5]	-	1.391	1.388	-	1.376	-

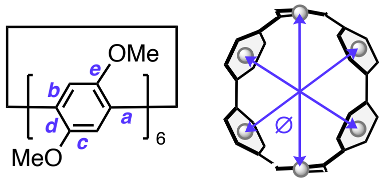

Supplementary Table 10 List of averaged harmonic oscillator model of aromaticity (HOMA) value of [6]CPP [4], [6]CPP-12OMe (**1**), and [6]CPP-12OMe dication ($\mathbf{1}^{2+}$).

Entry	HOMA value (avg.)
[6]CPP [4]	0.950
[6]CPP-12OMe (**1**)	0.895
[6]CPP-12OMe dication ($\mathbf{1}^{2+}$)	0.585

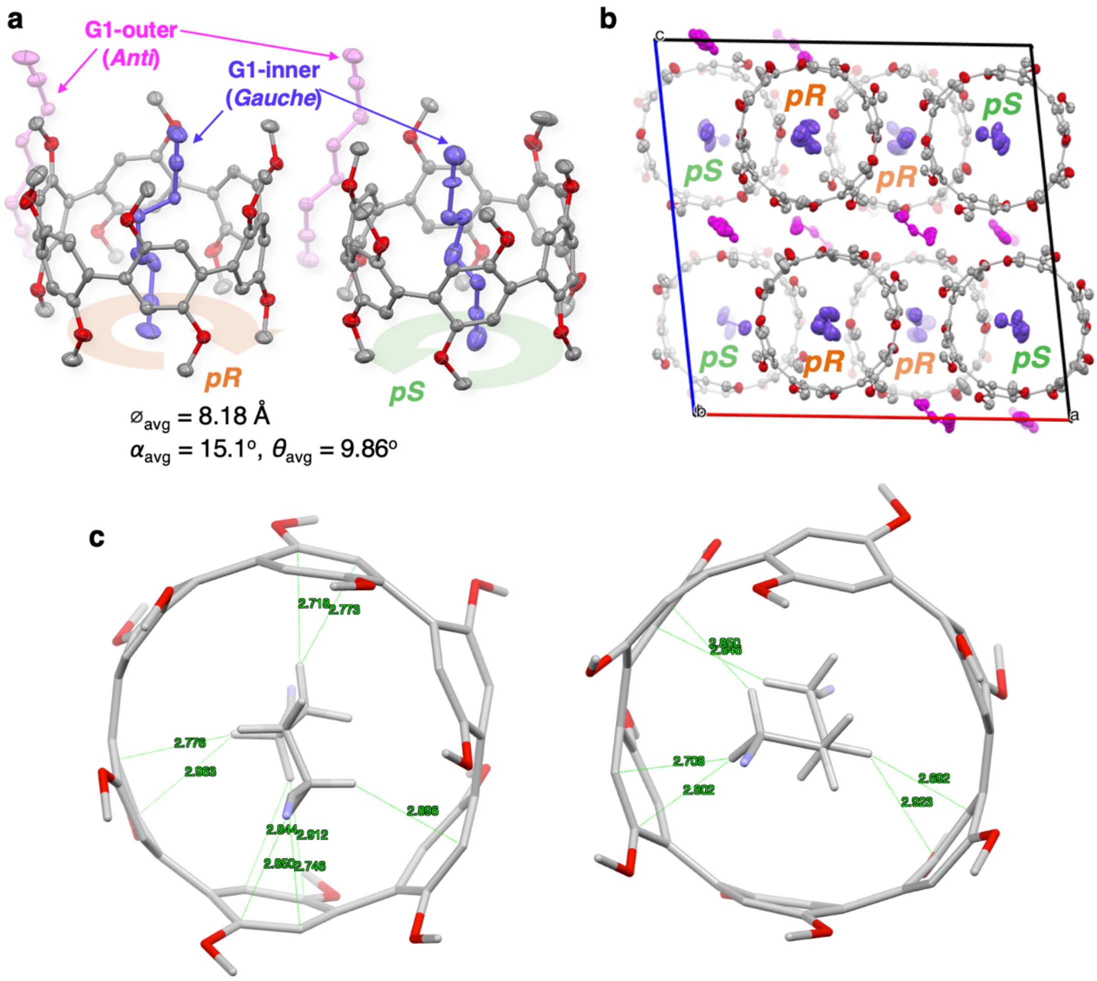

Supplementary Fig. 34 ORTEP drawing of inclusion complex (**1⊃G1**) (30% level of probability): (a) molecular structure (two isomers were analyzed as independent structures), (b) unit cell structure, (c) distance between the hydrogen atoms of the guest molecule (**G1**) and the carbon atoms of **1** in proximity within 3 Å.

Supplementary Table 11 Crystal and structure refinement data for macrocyclic Au complex (**Au-1**), [6]CPP-12OMe (**1**), dication (**1**$^{2+}$), and inclusion complex (**1⊃G1**).

	Au complex (**Au-1**)	[6]CPP-12OMe (**1**)	[6]CPP-12OMe Dication (**1**$^{2+}$)	Inclusion complex (**1⊃G1**)
CCDC	2222648	2222611	2222615	2222612
Solvent system	$CHCl_3$/acetone	1,2-DCE/hexane	1,2-DCE/hexane	$CHCl_3$/hexane
Color	colorless	red	green	purple
Formula	$C_{123}H_{186}Au_6O_{12}P_6$	$C_{48}H_{48}O_{12}$	$C_{48}H_{48}O_{12}$, $(Sb_{0.5}Cl_3)_4$	$C_{120}H_{128}N_8O_{24}$
F_w	3224.33	1373.72	1584.71	2066.37
Crystal system	trigonal	monoclinic	triclinic	monoclinic
Space group	$R\bar{3}$ (No. 148)	*C2/c* (No.15)	$P\bar{1}$ (No. 2)	$P2_1/c$ (No.14)
a (Å)	60.1043(11)	19.2027(4)	12.3025(2)	23.0840(5)
b (Å)	60.1043(11)	13.7646(3)	13.3818(2)	20.3445(4)
c (Å)	21.4163(4)	57.898(3)	20.1825(4)	23.1366(5)
α (deg)	90	90	94.240(7)	90
β (deg)	90	97.471(7)	91.979(7)	96.864(7)
γ (deg)	120	90	108.906(8)	90
V (Å^3)	67001.8	15173.5	3128.75	10787.8
Z	18	8	2	8
Dc (g cm^{-3})	1.438	1.203	1.682	2.544
No. of reflections measured	211884	86112	14716	101422
No. of unique reflections	27238	13834	9049	18961
No. of observations	19741 ($I > 2\sigma(I)$)	11937 ($I > 2\sigma(I)$)	8120 ($I > 2\sigma(I)$)	6475 ($I > 2\sigma(I)$)
R	0.0740	0.0458	0.0697	0.1048
R_w	0.1687	0.1216	0.1870	0.2401
GOF	1.041	1.032	0.952	0.951

Supplementary Methods

Supplementary Method 1: Synthesis of [Au_2Cl_2(dcpm)] (dcpm = bis(dicyclohexylphosphino)methane).[3] To a EtOH (39 mL) and H_2O (9 mL) solution of $HAuCl_4 \cdot 4H_2O$ (4.94 g, 12 mmol) was added tetrahydrothiophene (2.22 g, 25 mmol) at ambient temperature. The reaction mixture was stirred for 30 min at the same temperature. A white precipitate was formed during the reaction, which was collected by suction filtration and washed with EtOH (40 mL). The residue was dried under vacuum. [AuCl(tht)] (tht = tetrahydrothiophene) (3.56 g, 11 mmol, 93%) was obtained.

Then, to a CH_2Cl_2 solution (50 mL) of [AuCl(tht)] (3.22 g, 10 mmol) was added bis(dicyclohexylphosphino)methane (dcpm) (2.05 g, 5.0 mmol) at ambient temperature. After the reaction mixture was stirred for 30 min, Et_2O (150 mL) was added to the mixture. The precipitate was collected by suction filtration to give [Au_2Cl_2(dcpm)] (3.69 g, 4.2 mmol, 84%) as a white solid.

^{1}H NMR[3] (400 MHz, $CDCl_3$, 297 K): δ 2.21-2.18 (br, 4H, C_6H_{11}), 2.08 (t, 2H, J = 10.3 Hz), 2.05-1.97 (br, 8H, C_6H_{11}), 1.96-1.88 (br, 8H C_6H_{11}), 1.79-1.71 (br, 4H, C_6H_{11}), 1.54-1.21 (br, 20H, C_6H_{11}). ^{31}P{^{1}H} NMR[3] (202 MHz, $CDCl_3$, 297 K): δ 44.1 (s).

Supplementary Method 2: Synthesis of 2,2′,5,5′-tetramethoxy-1,1′-biphenyl (4).[6] A mixture of 1-bromo-2,5-dimethoxybenzene (1.75 g, 8.0 mmol), Cs_2CO_3 (7.83 g, 24 mmol), [Pd(PPh_3)$_4$] (0.47 g, 0.40 mmol), and 2,5-dimethoxyphenylboronic acid (1.75 g, 9.6 mmol) in degassed toluene/ethanol/water (75 mL/20 mL/20 mL) was stirred at 100 °C for 4 hours under argon atmosphere. After the mixture was allowed to cool to room temperature, toluene was added to the mixture and washed with water. The organic layer was dried over $MgSO_4$. The crude product was purified by silica gel column chromatography ($CHCl_3$, R_f = 0.35) to afford 2,2′,5,5′-tetramethoxy-1,1′-biphenyl (**4**) (1.98 g, 7.2 mmol, 90%) as a yellow solid.

^{1}H NMR[6] (400 MHz, $CDCl_3$, 297 K): δ 6.92 (d, 2H, J = 8.6 Hz, Ar-H), 6.87-6.83 (m, 4H, Ar-H), 3.78 (s, 6H, OCH_3), 3.73 (s, 6H, OCH_3).

Supplementary Method 3: Synthesis of 4,4′-dibromo-2,2′,5,5′-tetramethoxybiphenyl (3).[6] To a CH_2Cl_2 (40 mL) solution of 2,2',5,5'-tetramethoxy-1,1'-

biphenyl (**4**) (1.91 g, 7.0 mmol) was added NBS (3.74 g, 21.0 mmol), and stirred at 40 °C for 20 hours under argon atmosphere. After the mixture was allowed to cool to room temperature, the reaction mixture was washed with 10% $Na_2S_2O_3$ aq. (30 mL) and water (50 mL). The aqueous layer was extracted by CH_2Cl_2 (20 mL × 2). The combined organic layer was dried over $MgSO_4$. The crude product was purified by recrystallization ($CHCl_3$/MeOH = 10 mL/20 mL) to afford 4,4′-dibromo-2,2′,5,5′-tetramethoxybiphenyl (**3**) as a white solid (2.57 g, 6.0 mmol, 85%).

^{1}H NMR[6] (400 MHz, $CDCl_3$, 297 K): δ 7.17 (s, 2H, Ar-H), 6.82 (s, 2H, Ar-H), 3.85 (s, 6H, OCH_3), 3.73 (s, 6H, OCH_3).

Supplementary Method 4: Synthesis of (2,2′,5,5′-tetramethoxy-[1,1′-biphenyl]-4,4′-diyl)diboronic acid (L1).[7] To a THF solution (40 mL) of 4,4'-dibromo-2,2',5,5'-tetramethoxybiphenyl (**3**) (1.87 g, 4.3 mmol) was added *n*-BuLi (1.58 mol L^{-1} hexane solution, 8.2 mL, 12.9 mmol) at –78 °C, and stirred for 1 hour at the same temperature. Then, triisopropyl borate, B(O*i*-Pr)$_3$ (3.0 mL, 12.9 mmol) was added to the reaction mixture at −78 °C, and stirred for further 1 hour. After the mixture was stirred at room temperature for 1 hour, the reaction was quenched upon addition of water (10 mL) and 1 N HCl aq. (10 mL). The resulting solids were collected by suction filtration, rinsed with hexane, and dried in *vacuo* to afford (2,2′,5,5′-tetramethoxy-[1,1′-biphenyl]-4,4′-diyl)diboronic acid (**L1**) as a white solid (1.44 g, 4.0 mmol, 93%).

^{1}H NMR[7] (400 MHz, DMSO-d_6, 297 K): δ 7.77 (s, 4H, OH), 7.23 (s, 2H, Ar-H), 6.79 (s, 2H, Ar-H), 3.77 (s, 6H, OCH_3), 3.65 (s, 6H, OCH_3).

Supplementary Method 5: Crystallographic study. Single crystals of Au complex, $[Au_2(C_6H_4\text{-}2,5\text{-}OMe)_2(Cy_2PCH_2PCy_2)]_3$ (**Au-1**), were obtained by vapor diffusion of acetone into a $CHCl_3$ solution. Single crystals of [6]CPP-12OMe (**1**) and its dication (**1^{2+}**) were obtained by vapor diffusion of *n*-hexane into a 1,2-dichloroethane solution. Single crystals of inclusion complex (**1⊃G1**) were obtained by vapor diffusion of *n*-hexane into a $CHCl_3$ solution. The single X-ray structure determination was performed on a Rigaku RAPID2 (CuKα radiation, λ = 1.54178 Å). A numerical absorption correction (μ) was applied. The structure was solved by direct methods and refined by the full-matrix least-

squares method on F^2 with anisotropic temperature factors for non-hydrogen atoms.[8,9] All the hydrogen atoms were located at the calculated positions and refined with riding. The disordered guest molecules and/or methoxy groups were restricted by DFIX and SIMU for **Au-1**, **1**, and **1⊃G1**. The disordered counter anion was restricted by SIMU for $\mathbf{1}^{2+}$. The disordered solvent molecules were removed by SQUEEZE program for **Au-1** and **1**. ORTEP drawings are shown in Supplementary Figs. S31-34, and crystallographic data collection and refinement information are listed in Supplementary Table 11.

Single-crystal X-ray structures that contain the supplementary crystallographic data for this paper can be obtained free of charge from the Cambridge Crystallographic Data Centre at www.ccdc.cam.ac.uk/data_request/cif.

Supplementary Method 6: Computational details. Geometry optimization, relax potential energy surface scan, and CI-NEB[10] was carried out at the TPSS[11]-D3(BJ)[12,13]/def2-SVP[14] level of theory with the resolution of the identity (RI) approximation using the def2/J auxiliary basis set[15]. CI-NEB calculation was carried out with fully re-optimized geometry based on the relaxed potential energy surface scan. Kohn–Sham orbital was obtained at the B3LYP[16,17]/def2-SVP level of theory applied with the RIJCOSX[18] approximation using def2/J auxiliary basis set. TD-DFT calculation was done at the SOS-ωPBEPP86[19]/def2-SVP level of theory together with the RIJCOSX, RI-MP2/CIS(D), and Tamm-Dancoff approximation. The corresponding def2 series auxiliary basis set was used for RI approximation. Nucleus-independent chemical shifts (NICS(0)) and NMR chemical shifts values were calculated at the PBE0[20]/pcSseg-1[21] level of theory with the RIJCOSX approximation using an automatically generated auxiliary basis set implemented in ORCA[22]. Symmetry-adapted perturbation theory analysis was performed at the SAPT0[23]/jun-cc-pVDZ[24] level of theory[25]. ACID plot[26,27] was calculated at the CGST-PBE0/pcSseg-1 level of theory using AICD 3.0.4[27].

Molecular dynamics (MD) simulation was carried out by GFN-FF[28] level of theory using ORCA *via* interface to xTB. The initial geometry was searched using the CREST program at the GFN-FF level of theory. The most stable conformer was selected as the initial geometry. The system was equilibrated at 498 K for 5 ps with a time step of 0.2 fs and then cooled to 298 K for 1 ps with the same time step. The NVT ensemble with the

Berendsen thermostat[29] was employed for those simulations. Finally, the NVT simulation at 298 K with the Nosé–Hoover chain[30, 31] thermostat was conducted for 100 ps with a time step of 0.2 fs. The analytical linearized Poisson-Boltzmann (ALPB) solvation model for chloroform was used in MD calculation.

The computation was conducted using ORCA software[32,33] version 4.2.1 (geometry optimization, relaxed scan, and CI-NEB calculation), 5.0.1 (NICS calculation), 5.0.3 (TD-DFT, NMR chemical shifts, and MD calculation), PSI4 software[34] version 1.3.2 (SAPT calculation), Gaussian 09, revision E.01[35] (ACID calculation), xTB version 6.5.1[36] (MD calculation), CREST version 2.12[37] (conformer search), Multiwfn 3.8(dev) (QTAIM)[38], and NCIPLOT 4.2 (NCI plots)[39]. All the cartesian coordinates of geometry optimization, relaxed scan, and NEB calculation are available in a separate file with xyz format (Supplementally Data 1).

Supplementary Notes

Due to its high HOMO level (–4.41 eV), [6]CPP-12OMe (**1**) is easily photo-oxidized by oxygen in air or solvent, so the reaction and purification must be carried out under light-shielded conditions. [2]Rotaxane (**2**) is easily photo-oxidized by oxygen in air or solvent, so the reaction and purification must be carried out under light-shielded conditions.

Supplementary References

1. Segawa, Y., Omachi, H., & Itami, K. Theoretical studies on the structures and strain energies of cycloparaphenylenes. *Org. Lett.* **12**, 2262–2265 (2010).
2. (a) Toriumi, N. *et al.* In-plane aromaticity in cycloparaphenylene dications: a magnetic circular dichroism and theoretical study. *J. Am. Chem. Soc.* **137**, 82–85 (2015); (b) Kayahara, E., Kouyama, T., Kato, T. & Yamago, S. Synthesis and characterization of [*n*]CPP (n = 5, 6, 8, 10, and 12) radical cation and dications: size-dependent absorption, spin, and charge delocalization. *J. Am. Chem. Soc.* **138**, 338–344 (2016).
3. Tsuchido, Y., Abe, R., Ide, T. & Osakada, K. A macrocyclic Gold(I)–biphenylene complex: Triangular molecular structure with twisted Au_2(diphosphine) corners and reductive elimination of [6]cycloparaphenylene. *Angew. Chem. Int. Ed.* **59**, 22928–22932 (2020).
4. Xia, J. & Jasti, R. Synthesis, characterization, and crystal structure of [6]cycloparaphenylene. *Angew. Chem. Int. Ed.* **51**, 2474–2476 (2012).
5. Iuliucci, R. *et al.* Redetermination of 1,4-dimethoxybenzene. *Acta Crystallogr. Sect. E Struct. Rep. Online* **65**, o251 (2009).
6. C. R. K. Glasson *et al.* New nickel(ii) and iron(ii) helicates and tetrahedra derived from expanded quaterpyridines. *Dalton Trans.* **40**, 10481–10490 (2011).
7. Bushby, R. J. *et al.* *p*-Doped high spin polymers. *J. Mater. Chem.* **7**, 2343–2354 (1997).
8. (a) Sheldrick, G. M. Crystal Structure Refinement with SHELXL. *Acta Crystallogr. Sect. C Struct. Chem.* **71**, 3–8 (2015); (b) Sheldrick, G. M. A short history of *SHELX*. *Acta Crystallogr. Sect. A Found. Adv.* **64**, 112–122 (2008).
9. Dolomanov, O. V. *et al.* *OLEX2*: a complete structure solution, refinement and analysis program. *J. Appl. Cryst.* **42**, 339–341 (2009).
10. Ásgeirsson, V. *et al.* Nudged Elastic Band Method for Molecular Reactions Using Energy-Weighted Springs Combined with Eigenvector Following. *J. Chem. Theory Comput.* **17**, 4929–4945 (2021).
11. Tao, J., Perdew, J. P., Staroverov, V. N. & Scuseria, G. E. Climbing the Density Functional Ladder: Nonempirical Meta–Generalized Gradient Approximation Designed for Molecules and Solids. *Phys. Rev. Lett.* **91**, 146401 (2003).

12. Grimme, S., Antony, J., Ehrlich, S. & Krieg, H. A consistent and accurate ab initio parametrization of density functional dispersion correction (DFT-D) for the 94 elements H-Pu. *J. Chem. Phys.* **132**, 154104 (2010).
13. Grimme, S., Ehrlich, S. & Goerigk, L. Effect of the damping function in dispersion corrected density functional theory. *J. Comput. Chem.* **32**, 1456–1465 (2011).
14. Weigend, F. & Ahlrichs, R. Balanced basis sets of split valence, triple zeta valence and quadruple zeta valence quality for H to Rn: Design and assessment of accuracy. *Phys. Chem. Chem. Phys.* **7**, 3297–3305 (2005).
15. Weigend, F. Accurate Coulomb-fitting basis sets for H to Rn. *Phys. Chem. Chem. Phys.* **8**, 1057–1065 (2006).
16. Becke, A. D. Density-functional thermochemistry. III. The role of exact exchange. *J. Chem. Phys.* **98**, 5648–5652 (1993).
17. Stephens, P. J., Devlin, F. J., Chablowski, C. F. & Frisch, M. J. Ab Initio Calculation of Vibrational Absorption and Circular Dichroism Spectra Using Density Functional Force Fields. *J. Phys. Chem.* **98**, 11623–11627 (1994).
18. Helmich-Paris, B., de Souza, B., Neese, F. & Izsák, R. An improved chain of spheres for exchange algorithm. *J. Chem. Phys.* **155**, 104109 (2021).
19. Casanova-Páez, M. & Goerigk, L. Time-Dependent Long-Range-Corrected Double-Hybrid Density Functionals with Spin-Component and Spin-Opposite Scaling: A Comprehensive Analysis of Singlet–Singlet and Singlet–Triplet Excitation Energies. *J. Chem. Theory Comput.* **17**, 5165–5186 (2021).
20. Adamo, C. & Barone, V. Toward reliable density functional methods without adjustable parameters: The PBE0 model. *J. Chem. Phys.* **110**, 6158–6170 (1999).
21. Jensen, F. Segmented Contracted Basis Sets Optimized for Nuclear Magnetic Shielding. *J. Chem. Theory Comput.* **11**, 132–138 (2015).
22. Stoychev, G. L., Auer, A. A. & Neese, F. Automatic Generation of Auxiliary Basis Sets. *J. Chem. Theory Comput.* **13**, 554–562 (2017).
23. Żuchowski, P. S., Podeszwa, R., Moszyński, R., Jeziorski, B. & Szalewicz K., Symmetry-adapted perturbation theory utilizing density functional description of monomers for high-spin open-shell complexes. *J. Chem. Phys.* **129**, 084101 (2008).

24. Papajak, E., Zheng, J., Xu, X., Leverentz, H. R. & Truhlar, D. G. Perspectives on Basis Sets Beautiful: Seasonal Plantings of Diffuse Basis Functions. *J. Chem. Theory Comput.* **7**, 3027–3034 (2011).
25. Parker, T. M., Burns, L. A., Parrish, R. M., Ryno, A. G. & Sherrill, C. D. Levels of symmetry adapted perturbation theory (SAPT). I. Efficiency and performance for interaction energies. *J. Chem. Phys.* **140**, 094106 (2014).
26. Herges, R. & Geuenich, D. Delocalization of Electrons in Molecules. *J. Phys. Chem. A* **105**, 3214–3220 (2001).
27. Geuenich, D., Hess, K., Koehler, F. & Herges, R. Anisotropy of the Induced Current Density (ACID), a General Method To Quantify and Visualize Electronic Delocalization. *Chem. Rev.* **105**, 3758–3772 (2005).
28. Spicher, S. & Grimme, S. Robust Atomistic Modeling of Materials, Organometallic, and Biochemical Systems. *Angew. Chem. Int. Ed.* **59**, 15665–15673 (2020).
29. Berendsen, H. J. C., Postma, J. P. M., van Gunsteren, W. F., DiNola, A. & Haak, J. R. Molecular dynamics with coupling to an external bath. *J. Chem. Phys.* **81**, 3684–3690 (1984).
30. Nosé, S. A unified formulation of the constant temperature molecular dynamics methods. *J. Chem. Phys.* **81**, 511–519 (1984).
31. Hoover, W. G. Canonical dynamics: Equilibrium phase-space distributions. *Phys. Rev. A*, **31**, 1695–1697 (1985).
32. Neese, F. Software update: the ORCA program system, version 4.0. *WIREs Comput. Mol. Sci.* **8**, e1327 (2018).
33. Neese, F., Wennmohs, F., Becker, U. & Riplinger, C. The ORCA quantum chemistry program package. *J. Chem. Phys.* **152**, 224108 (2020).
34. Parrish, R. M. *et al.* Psi4 1.1: An Open-Source Electronic Structure Program Emphasizing Automation, Advanced Libraries, and Interoperability. *J. Chem. Theory Comput.* **13**, 3185–3197 (2017).
35. Gaussian 09, Revision E.01, Frisch, M. J. *et al*. Gaussian, Inc., Wallingford CT, 2009.
36. Bannwarth, C. *et al*. Extended tight-binding quantum chemistry methods. *WIREs Comput. Mol. Sci.* **11**, e01493 (2021).

37. Pracht, P., Bohle, F. & Grimme, S. Automated exploration of the low-energy chemical space with fast quantum chemical methods. *Phys. Chem. Chem. Phys.* **22**, 7169–7192 (2020).
38. Lu, T. & Chen, F. Multiwfn: A multifunctional wavefunction analyzer. *J. Comput. Chem.* **33**, 580–592 (2012).
39. (a) Boto, R. A. *et al*. NCIPLOT4: A new step towards a fast quantification of noncovalent interactions, https://github.com/juliacontrerasgarcia/nciplot; (b) Johnson, E. R. *et al*. Revealing noncovalent interactions, *J. Am. Chem. Soc.* **132**, 6498–6506 (2010); (c) Contreras-Garcia, J. *et al*. NCIPLOT: A program for plotting noncovalent interaction regions, *J. Chem. Theory Comput.* **7**, 625–632 (2011).
