# Supplementary material for: A dodecamethoxy[6]cycloparaphenylene consisting entirely of hydroquinone ethers: unveiling in-plane aromaticity through a rotaxane structure
Source: Nat Commun. 2023 Dec 7;14:8091. doi: 10.1038/s41467-023-43907-7 (PMC10703805; doi:10.1038/s41467-023-43907-7)
Supplement: Supplementary file 3 — Description of Additional Supplementary Files [file 41467_2023_43907_MOESM3_ESM.pdf]

Description of Additional Supplementary Files

Supplementary Data 1: Cartesian coordinates of the calculated structures.

Supplementary Movie 1: Molecular dynamics (MD) simulations of the shuttling movement of [2]rotaxane.
